# Bioinformatics and system biology approach to identify the influences among COVID-19, influenza, and HIV on the regulation of gene expression

**DOI:** 10.3389/fimmu.2024.1369311

**Published:** 2024-03-27

**Authors:** Zhen Zhang, Hao Jin, Xu Zhang, Mei Bai, Kexin Zheng, Jing Tian, Bin Deng, Lingling Mao, Pengcheng Qiu, Bo Huang

**Affiliations:** ^1^ Microbiology Laboratory Department, Jinzhou Center for Disease Control and Prevention, Jinzhou, Liaoning, China; ^2^ Department of Immunology, School of Basic Medical Science, Jinzhou Medical University, Jinzhou, Liaoning, China; ^3^ Laboratory Department, Jinzhou Central Hospital, Jinzhou, Liaoning, China; ^4^ Institute for Prevention and Control of Infection and Infectious Diseases, Liaoning Provincial Center for Disease Control and Prevention, Shenyang, Liaoning, China; ^5^ Thoracic Surgery Department, The First Affiliated Hospital of Jinzhou Medical University, Jinzhou, Liaoning, China; ^6^ Thoracic Surgery Department, Yingkou Central Hospital, Yingkou, Liaoning, China

**Keywords:** COVID-19, influenza, HIV, differentially expressed genes, immune infiltration, hub genes, protein-protein interaction networks, drug chemicals

## Abstract

**Background:**

Coronavirus disease (COVID-19), caused by SARS-CoV-2, has emerged as a infectious disease, coexisting with widespread seasonal and sporadic influenza epidemics globally. Individuals living with HIV, characterized by compromised immune systems, face an elevated risk of severe outcomes and increased mortality when affected by COVID-19. Despite this connection, the molecular intricacies linking COVID-19, influenza, and HIV remain unclear. Our research endeavors to elucidate the shared pathways and molecular markers in individuals with HIV concurrently infected with COVID-19 and influenza. Furthermore, we aim to identify potential medications that may prove beneficial in managing these three interconnected illnesses.

**Methods:**

Sequencing data for COVID-19 (GSE157103), influenza (GSE185576), and HIV (GSE195434) were retrieved from the GEO database. Commonly expressed differentially expressed genes (DEGs) were identified across the three datasets, followed by immune infiltration analysis and diagnostic ROC analysis on the DEGs. Functional enrichment analysis was performed using GO/KEGG and Gene Set Enrichment Analysis (GSEA). Hub genes were screened through a Protein-Protein Interaction networks (PPIs) analysis among DEGs. Analysis of miRNAs, transcription factors, drug chemicals, diseases, and RNA-binding proteins was conducted based on the identified hub genes. Finally, quantitative PCR (qPCR) expression verification was undertaken for selected hub genes.

**Results:**

The analysis of the three datasets revealed a total of 22 shared DEGs, with the majority exhibiting an area under the curve value exceeding 0.7. Functional enrichment analysis with GO/KEGG and GSEA primarily highlighted signaling pathways associated with ribosomes and tumors. The ten identified hub genes included *IFI44L*, *IFI44*, *RSAD2*, *ISG15*, *IFIT3*, *OAS1*, *EIF2AK2*, *IFI27*, *OASL*, and *EPSTI1*. Additionally, five crucial miRNAs (hsa-miR-8060, hsa-miR-6890-5p, hsa-miR-5003-3p, hsa-miR-6893-3p, and hsa-miR-6069), five essential transcription factors (CREB1, CEBPB, EGR1, EP300, and IRF1), and the top ten significant drug chemicals (estradiol, progesterone, tretinoin, calcitriol, fluorouracil, methotrexate, lipopolysaccharide, valproic acid, silicon dioxide, cyclosporine) were identified.

**Conclusion:**

This research provides valuable insights into shared molecular targets, signaling pathways, drug chemicals, and potential biomarkers for individuals facing the complex intersection of COVID-19, influenza, and HIV. These findings hold promise for enhancing the precision of diagnosis and treatment for individuals with HIV co-infected with COVID-19 and influenza.

## 1 Introduction

The contagious illness, known as Coronavirus disease (COVID-19), is generated by the Severe Acute Respiratory Syndrome Coronavirus 2 (SARS-CoV-2) virus (1, 2) and emerged in Wuhan, China, in December 2019, resulting in significant casualties, severe consequences, and a significant menace to public health, food systems, and the global workforce (1–4).According to the World Health Organization (WHO), typical symptoms of COVID-19 encompass fever, diarrhea, sore throat, dry cough, fatigue, and musculoskeletal manifestations such as joint and muscle pain (5–9). Respiratory tract infection generated by influenza virus occurs through direct infection of respiratory epithelial cells, leading to both Innate and adaptive immune responses are activated. Indeed, blocking the transmission of flu viruses is essential (10), given that the onset of the flu season can substantially impact human well-being. Influenza, a prevalent respiratory pathogen, causes regular outbreaks and occasional severe epidemics globally (11).

As of 2021, the WHO estimates that approximately 36.3 million individuals have lost their lives due to human immunodeficiency virus (HIV)/acquired immunodeficiency syndrome (AIDS), with a global population of 37.7 million affected by this disease. HIV results in HIV infection and AIDS (12), characterized by the blood is described by a deficiency of CD4 T cells, with a count below 200 cells per liter or the presence of AIDS-defining illnesses (13, 14). Earlier research has indicated that individuals with HIV face a twofold enhance in the probability of succumbing to COVID-19 and experiencing more adverse outcomes related to COVID-19 (15–17). Recent findings suggest that HIV is an separate indicator of heightened risk for severe/critical COVID-19 and mortality during hospitalization. Although manifestations of influenza infection are comparable between patients with and without HIV, HIV-positive individuals appear more susceptible to complications from lower respiratory tract disease (18). Mortality rates among people living with HIV after contracting influenza have decreased due to antiretroviral treatment but remain higher compared to individuals without HIV (19). Numerous researches have examined COVID-19 and influenza-infected persons, covering transmission methods, clinical characteristics, immune response patterns, symptoms, laboratory tests, radiological indications, morbidity, and mortality rates (10, 20–22).

Notably, individuals afflicted with COVID-19 reveal symptoms akin to those observed in influenza patients, including cough, pneumonia, acute respiratory distress syndrome (ARDS), fever, imbalanced immune response, excessive inflammation, depletion and dysfunction of T cells, and immune evasion mechanisms (21–23). SARS-CoV-2 exhibits a higher transmissibility than seasonal influenza, although the latter has a significantly lower fatality rate (21, 22). It has also been established that COVID-19 individuals are more susceptible to chemosensory dysfunction, rash, and reproductive system damage than those with influenza (21, 24).

**Figure 1:**
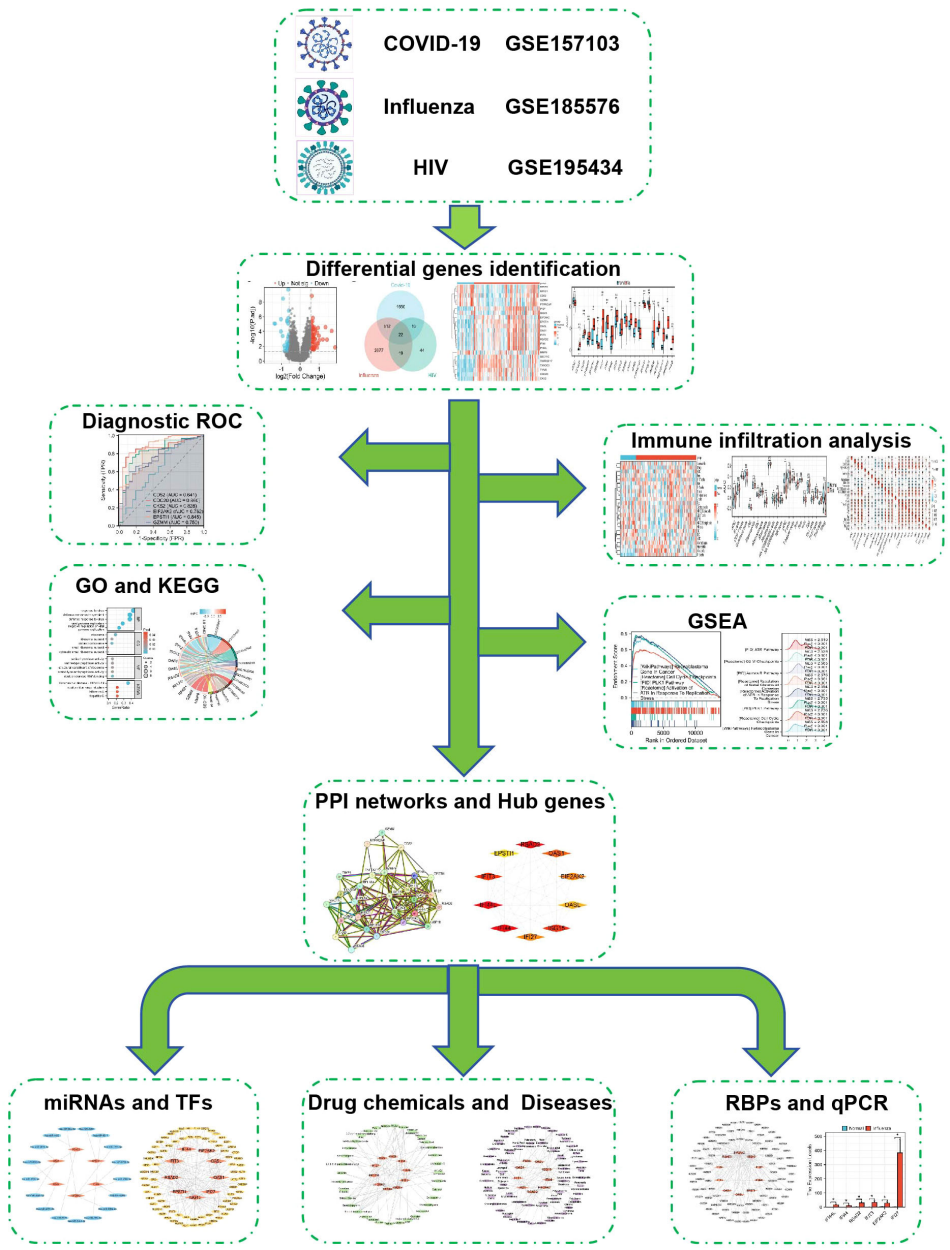
Schematic illustration of the overall general workflow of this study.

This research employed three datasets to uncover biological connections among COVID-19, influenza, and HIV. Shared DEGs were identified, followed by immune infiltration and diagnostic Receiver Operating Characteristic (ROC) curve analyses. Functional enrichment analysis and identification of potential biological pathways were performed using Gene Ontology (GO)/Kyoto Encyclopedia of Genes and Genomes (KEGG) and GSEA methods. PPIs were utilized to analyze shared DEGs and identify hub genes. Based on hub genes, several aspects were analyzed, including microRNAs (miRNAs), transcription factors (TFs), drug chemicals, diseases, RNA-binding proteins (RBPs), and expression verification of some hub genes by qPCR. **Figure 1** illustrates the sequential workflow during the study. Abbreviation and full name comparison table can be found in **Supplementary Table 1**.

## 2 Materials and methods

### 2.1 Data collection

When selecting specific sequencing datasets, we carefully evaluated factors such as data accessibility, data quality, disease relevance, and consistency with study objectives. In order to explore common genetic interactions and potential therapeutic targets among COVID-19, influenza, and HIV, we acquired microarray and RNA-seq data from the Gene Expression Omnibus (GEO) database, which is administered by the National Center for Biotechnology Information (NCBI).The COVID-19 dataset (GEO accession ID: GSE157103) comprised 126 samples (100 COVID-19 and 26 non-diseased control samples) obtained from whole blood, and transcriptional analysis was performed. RNA sequencing analysis of GSE157103 utilized the Illumina NovaSeq 6000 (Homo sapiens) platform (GPL24676) for high-throughput sequencing-based expression profiling. The influenza dataset (GEO accession ID: GSE185576) included 152 samples of whole blood from 127 influenza-positive cases and 25 healthy controls. The dataset was created using the Agilent SurePrint G3 platform (GPL21185), and expression profiling analysis was conducted using array-based methods. Similarly, the HIV dataset (GEO accession ID: GSE195434) involved whole blood gene expression profiles from 90 samples, including 69 HIV-infected and 21 HIV-uninfected individuals. Expression profiling utilized the Illumina HumanHT-12 platform (GPL10558), and analysis was performed through array-based methods.

### 2.2 DEGs detection and shared DEGs among COVID-19, influenza, and HIV

Our research primarily focused on identifying DEGs in the COVID-19, Influenza, and HIV datasets. For the COVID-19 dataset, sequencing data were initially obtained from GEO. The Xiantao online tool’s differential analysis transcriptome-counts module was utilized for standardization, and the resulting DEG data were downloaded. In the case of the influenza dataset, the GEO2R web tool (https://www.ncbi.nlm.nih.gov/geo/geo2r/) was employed to analyze DEGs, utilizing the Xiantao online tool for DEG identification. Similarly, the Xiantao tool was utilized for the analysis of the HIV dataset and identify DEGs. DEGs showing an adjusted p-value was less than 0.05 and |log2FC| > 0.58 were regarded statistically significant and used for subsequent analysis. To visualize the overlapping up-regulated and down-regulated DEGs across all three datasets, three Venn diagrams were generated: one for the intersection of all DEGs, one for the intersection of only up-regulated DEGs, and another for the intersection of only down-regulated DEGs. Additionally, volcano plots were generated for each dataset to visualize the differential genes. Heat maps and group comparison diagrams, derived from the 22 identified DEGs, were created to represent the three datasets. All these visualizations, including volcano plots, Venn diagrams, heat maps, and group comparison diagrams, were generated using the Xiantao tool.

### 2.3 Diagnostic ROC curve analysis of 22 DEGs among COVID-19, influenza, and HIV

In our study, we utilized grouped data and expression data of 22 differential genes to generate ROC curves for each of the three datasets. Each dataset drew 4 graphs respectively, of which the first two graphs had 6 genes, and the last two graphs contained 5 genes, 12 pictures.

### 2.4 Immune infiltration analysis in COVID-19, influenza and HIV datasets

In our analysis, we assessed each sample’s extent of immune cell infiltration by utilizing the Immune Infiltration ssGSEA algorithm module of the Xiantao tool. This approach involved employing single-sample gene set enrichment analysis (ssGSEA) to assess the extent of immune cell presence. Across the three datasets, we utilized the Xiantao online tool to generate heat maps, group comparison maps, and correlation heat maps, incorporating information on 24 different immune cells.

### 2.5 Functional enrichment analysis

Analysis of functional enrichment includes GO analysis, a widely used approach (25) that categorizes genes into the three main domains: biological process (BP), cellular composition (CC), and molecular function (MF). Additionally, substantially enriched pathways were identified using KEGG pathway analysis, providing valuable insights into the biological significance of genomic data (26). For the GO/KEGG data analysis, we focused on 22 genes that exhibited differential expression. Initially, a screening process was implemented, wherein the criteria for inclusion involved a false discovery rate (FDR) of less than 0.25 and an adjusted p-value of lower than 0.05. The top 5 terms for BP, CC, and MF (KEGG had the top 4) were observed in a bubble graph. Subsequently, we screened the top 3 results for BP, CC, MF, and KEGG to construct a graphical representation of the network. To further explore the connections between GO/KEGG terms and logFC across the three datasets, we utilized the 22 DEGs to generate chord and circle diagrams.

### 2.6 GSEA of common DEGs among COVID-19, influenza, and HIV

In our study, we conducted GSEA using all samples from the three datasets. Upon obtaining the data, we screened pathways based on a FDR of less than 0.25 and an adjusted p-value of lower than 0.05. Subsequently, we sorted the pathways in descending order based on Normalized Enrichment Score (NES) values and selected 8 important pathways for each dataset. To visualize the results, we generated two classic graphs for each dataset. Each classic graph contained information on 4 pathways. Additionally, we generated a mountain plot for each dataset, illustrating all 8 selected pathways. The classic graphs and mountain plots were created using the Xiantao tool, providing a comprehensive visualization of the enriched pathways and their significance in the context of the analyzed datasets.

### 2.7 PPIs and hub genes among COVID-19, influenza, and HIV

In our study, we identified the PPIs of shared DEGs among COVID-19, Influenza, and HIV. The connections between various diseases based on protein interactions were explored utilizing the STRING database’s search tool (version 12.0, https://cn.string-db.org/) (27). The STRING database consolidates established and anticipated connections among proteins, encompassing both physical interactions and functional relationships. For the construction of the PPIs, we set a minimum interaction score of 0.150 as the low confidence level and established a limit of a maximum of 5 interactors in the 1st shell. Additionally, we implemented a minimum required interaction score of 0.900 as the highest confidence level. We also limited the maximum number of interactors to include only non-query proteins in the 1st shell, resulting in the generation of an additional PPIs for common DEGs. To enhance the visualization and facilitate further PPIs studies, we utilized Cytoscape software (version 3.9.1) (28). In the identification of hub genes within the PPIs, a plugin for Cytoscape called Cytohubba was employed. We employed five algorithms within Cytohubba, namely MCC, DMNC, MNC, Degree, and EPC, to screen for hub genes (29). Using various algorithms, the top 10 hub genes were chosen. Afterwards, the production of a Venn diagram was conducted to determine the genes that overlap among these algorithms, leading to the identification of core hub genes.

### 2.8 The regulatory interaction network of hub genes, which were interconnected with miRNAs and TFs

MiRNAs are small, naturally occurring, non-coding RNAs that function by binding to gene transcripts, influencing protein expression (30). Transcription factors are essential regulators of transcription rates, binding to specific genes (31) and providing valuable molecular insights. To identify putative Hub genes-miRNAs, we employed the miRWalk database (version 3.0, http://mirwalk.umm.uni-heidelberg.de/) and considered pairings with a number greater than or equal to 20. Additionally, the miRDB database (version 6.0, https://mirdb.org/) was used for this screening process. Genes identified by both databases were overlapped to acquire the Hub genes-miRNAs. Subsequently, these selected genes were imported into Cytoscape software to generate a graphical representation of the network. For Hub genes-TFs, we utilized the database of hTFtarget (http://bioinfo.life.hust.edu.cn/hTFtarget#!/),and the database of ChIPBase (version 3.0, https://rnasysu.com/chipbase3/index.php). Genes obtained from both databases were overlapped to select the Hub genes-TFs. Subsequently, the selected genes were imported into the Cytoscape software platform in order to generate a network diagram.

### 2.9 The network of regulatory interactions between hub genes and chemicals, as well as hub genes and diseases

To predict the interaction between proteins and drugs and recognize drug molecules based on target genes, we utilized the networkanalyst database (https://www.networkanalyst.ca/) and the Comparative Toxicogenomics Database (CTD: https://ctdbase.org/). Specifically, we aimed to identify the small molecule structures of 10 hub genes with a Reference Count of 2 or more. Genes filtered by both databases were compared to identify intersecting hub genes associated with chemicals. Subsequently, these hub genes associated with chemicals were imported into Cytoscape software to generate a network diagram. In addition, we investigated the connection between Hub genes and diseases to identify related diseases with common hub genes. Initially, we used the DisGeNET database (version 7.0, http://www.disgenet.org/) and the MalaCards database (Version 5.17, https://www.malacards.org/) to screen for related genes. Genes extracted from the two databases were then compared to identify Hub genes-disease relationships. The identified genes were imported into Cytoscape software, where a network diagram was constructed.

### 2.10 Prediction of hub genes–RBPs and qPCR verification

To identify hub genes–RBPs, we utilized the ENCORI database (https://rnasysu.com/encori/). The screening process involved selecting genes with several supported CLIP-seq experiments greater than 1. The selected hub genes–RBPs were then filtered, and the selected genes were integrated into the Cytoscape application to generate a graphical representation of the network, providing insights into potential RNA-protein interactions.

For gene expression verification, the qPCR method was employed on the datasets: COVID-19, influenza, and HIV. For COVID-19 testing, positive samples were patient throat swab samples, and negative control samples were oral mucosal cells from a healthy person. For influenza and HIV testing, positive samples were patient whole blood samples, and negative control samples were whole blood samples from a healthy person. Experimental samples were collected from the First Affiliated Hospital of Jinzhou Medical University. RNA extraction was performed on oral mucosal cells and whole blood samples utilizing the Viral Nucleic Acid Extraction Kit (SDK60104, Jiangsu Bioperfectus) and quantified using a spectrophotometer (NP80, Implen). The reverse transcription of RNA into cDNA was performed employing ReverTra Ace^®^ qPCR RT Master Mix with gDNA Remover (FSQ-301, TOYOBO). Real-time quantitative PCR (qPCR) was conducted utilizing Tag Pro Universal SYBR qPCR Master Mix (Q712-02, Vazyme) and a real-time PCR system (QS7, ABI). In the experimental setup for all three diseases, including COVID-19, influenza, and HIV, each disease was tested using 4 positive and 4 negative samples. The experiment was conducted with each sample comprising 3 sub-wells for robustness and reliability. The primer sequences utilized for qPCR can be discovered in **Supplementary Table 2**.

For the COVID-19 trial, three genes (*IFIT3*, *EIF2AK2*, and *IFI27*) were selected; for the influenza trial, six genes (*IFI44L*, *IFI44*, *RSAD2*, *IFIT3*, *EIF2AK2*, and *IFI27*) were chosen; and for the HIV trial, seven genes (*IFI44L*, *IFI44*, *RSAD2*, *IFIT3*, *EIF2AK2*, *IFI27*, and *ISG15*) were included. After qPCR amplification, the cycle threshold (CT) values of each sample were recorded in Excel and then imported into the Xiantao online tools for further analysis. The 2^-ΔΔCt^ approach, a widely used method in qPCR data analysis, was employed to calculate the relative expression levels for each sample. Subsequently, a histogram was generated to visually represent the relative expression levels of the selected genes across positive and negative samples for each disease.

### 2.11 Complex interrelationships of hub gene, miRNA, transcription factor, drug chemical, disease, and RBP

By considering 10 hub genes, 5 predicted significant miRNAs, 5 predicted transcription factors, 10 predicted drug chemicals, 10 predicted diseases, and 5 predicted RNA-binding proteins, we constructed a comprehensive network diagram to visually represent the mutual regulatory relationships among these six distinct types of molecules.

### 2.12 Statistical analysis

The statistical analysis and visualization were conducted employing the Xiantao online tool (https://www.xiantaozi.com/). The group comparison diagrams were employed to analyze DEGs, utilizing the Wilcoxon rank sum test. Additionally, the Spearman correlation coefficient was utilized to assess the relationships between immune cells. Statistical significance extents were defined as p < 0.05 (*), p < 0.01 (**), and p < 0.001 (***), while ‘ns’ indicated no significant difference.

## 3 Result

### 3.1 Detecting DEGs and identifying shared DEGs for COVID-19, influenza, and HIV

**Figure 2:**
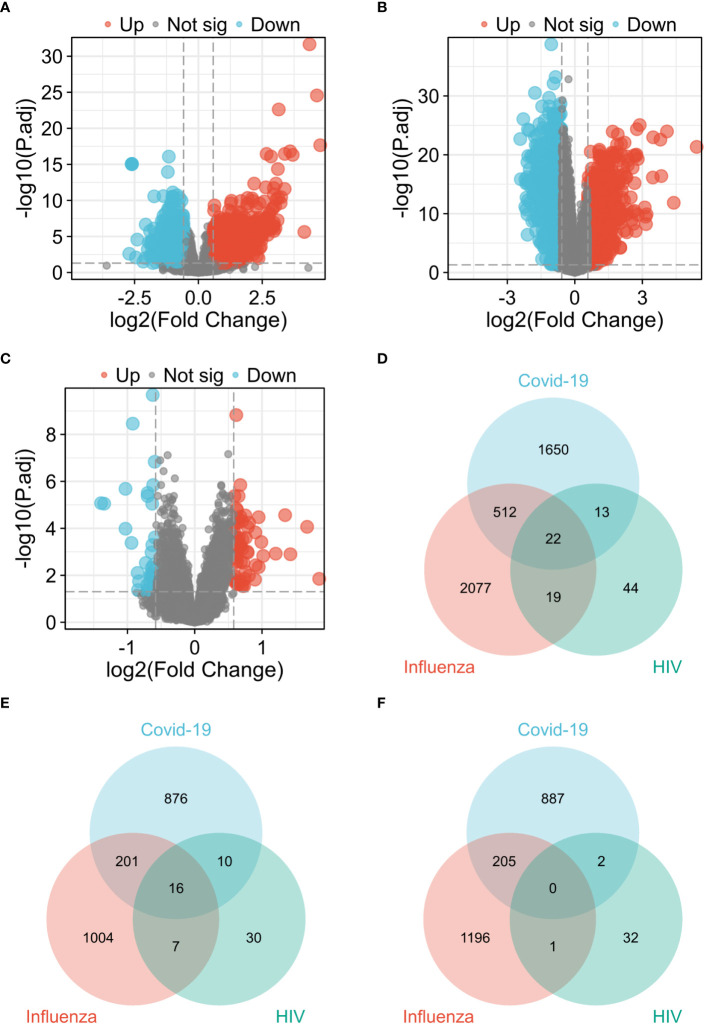
Visualization of common diferentially expressed genes (DEGs) among COVID-19, Influenza and HIV. **(A)** Volcano plot of COVID-19 in GSE157103 dataset. **(B)** Volcano plot of Influenza in GSE185576 dataset. **(C)** Volcano plot of HIV in GSE195434 dataset. **(D)** Venn diagram showing the overlap of up-regulated and down-regulated DEGs among three diseases. **(E)** Venn diagram showing only up-regulated DEGs overlap among three diseases. **(F)** Venn diagram showing only down-regulated DEGs overlap among three diseases.

In the COVID-19 dataset GSE157103, 2,197 genes exhibiting differential expression were detected, comprising of 1,103 up-regulated genes and 1,094 down-regulated genes. Similarly, in the influenza dataset GSE185576, 2,630 DEGs (1,228 up-regulated and 1,402 down-regulated) were discovered, while the HIV dataset GSE195434 revealed 98 DEGs (63 up-regulated and 35 down-regulated). **Figure 2** presents three volcano plots, specifically **Figure 2A** for COVID-19, **Figure 2B** for Influenza, and **Figure 2C** for HIV, effectively delineating the overall gene expression patterns. Red dots indicate up-regulated genes, while blue dots display down-regulated genes. By generating a Venn diagram, we identified 22 shared differential genes among COVID-19, Influenza, and HIV (**Figure 2D**). Among these genes, 16 were up-regulated DEGs (**Figure 2E**), with no observed down-regulated DEGs (**Figure 2F**). For the remaining 6 genes, 5 of them (*CD52*, *GZMM*, *PTPRCAP*, *RPLP0*, *RPS21*) were down-regulated in the COVID-19 and influenza datasets, and up-regulated in the HIV dataset; the other gene, *MMP9*, was down-regulated in the COVID-19 and HIV datasets, and upregulated in the influenza dataset. A total of 22 DEGs can be found in **Supplementary Table 3**.

**Figure 3:**
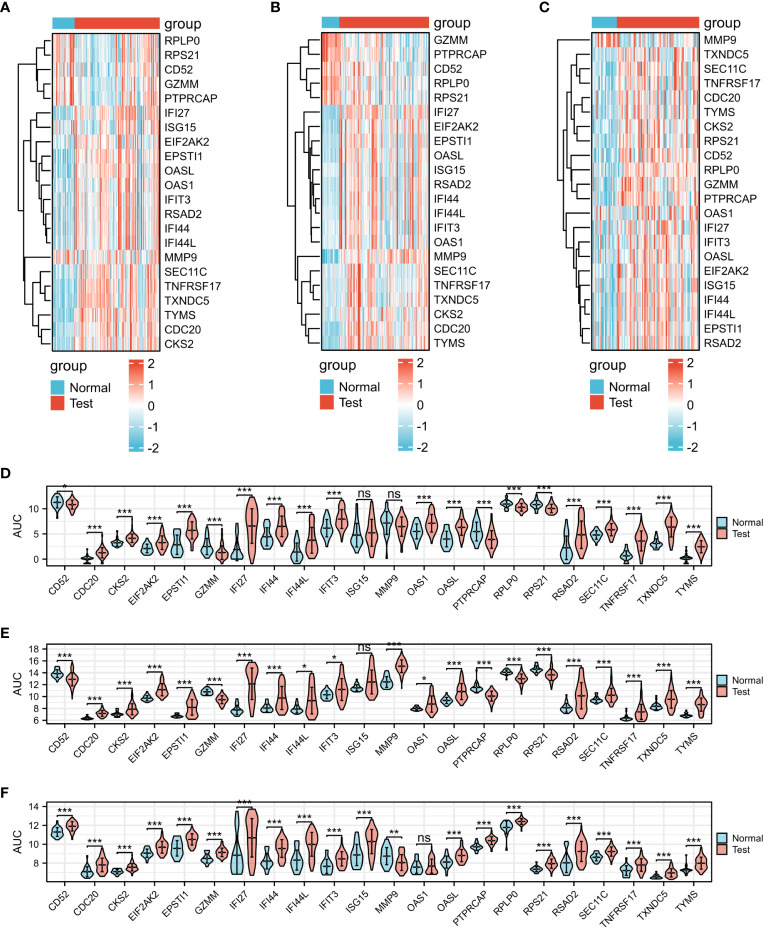
Expression analysis of the 22 DEGs among three diseases. **(A)** Heat map of COVID-19. **(B)** Heat map of Influenza. **(C)** Heat map of HIV. **(D)** mRNA expression levels of COVID-19. **(E)** mRNA expression levels of Influenza. **(F)** mRNA expression levels of HIV.

The three presented heat maps enable visualization of the comprehensive expression patterns of the 22 differential genes across COVID-19 (**Figure 3A**), Influenza (**Figure 3B**), and HIV (**Figure 3C**). Our analysis involved the examination of 22 DEGs, comparing the gene expression variations within the disease groups of COVID-19, Influenza, and HIV against their respective healthy groups. Within the context of the COVID-19 test, the control group exhibited no statistically significant difference in expression levels of *ISG15* and *MMP9*. However, the remaining 20 genes displayed noteworthy variations (**Figure 3D**). In the Influenza test, only the gene *ISG15* did not manifest a notable distinction between the group with the disease and those in good health, while the remaining 21 genes exhibited substantial disparities (**Figure 3E**). Simultaneously, during the HIV test, only one gene, *OAS1*, demonstrated no significant difference, whereas the other 21 genes displayed significant variations (**Figure 3F**).

### 3.2 Diagnostic ROC curve analysis of 22 DEGs among COVID-19, influenza, and HIV

**Figure 4:**
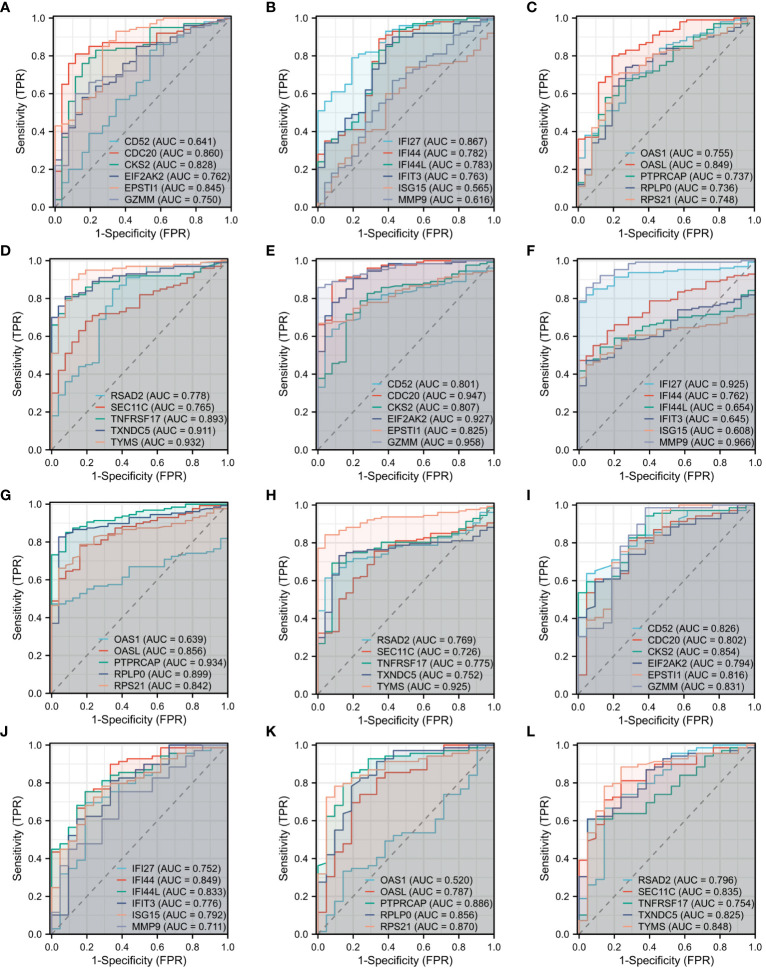
Diagnostic ROC curve analysis of 22 DEGs among three diseases. **(A)** ROC of CD52, CDC20, CKS2, EIF2AK2, EPSTI1, GZMM in COVID-19. **(B)** ROC of IFI27, IFI44, IFI44L, IFIT3, ISG15, MMP9 in COVID-19. **(C)** ROC of OAS1, OASL, PTPRCAP, RPLP0, RPS21 in COVID-19. **(D)** ROC of RSAD2, SEC11C, TNFRSF17, TXNDC5, TYMS in COVID-19. **(E)** ROC of CD52, CDC20, CKS2, EIF2AK2, EPSTI1, GZMM in Influenza. **(F)** ROC of IFI27, IFI44, IFI44L, IFIT3, ISG15, MMP9 in Influenza. **(G)** ROC of OAS1, OASL, PTPRCAP, RPLP0, RPS21 in Influenza. **(H)** ROC of RSAD2, SEC11C, TNFRSF17, TXNDC5, TYMS in Influenza. **(I)** ROC of CD52, CDC20, CKS2, EIF2AK2, EPSTI1, GZMM in HIV. **(J)** ROC of IFI27, IFI44, IFI44L, IFIT3, ISG15, MMP9 in HIV. **(K)** ROC of OAS1, OASL, PTPRCAP, RPLP0, RPS21 in HIV. **(L)** ROC of RSAD2, SEC11C, TNFRSF17, TXNDC5, TYMS in HIV.

The diagnostic efficacy of the 22 DEGs in COVID-19, influenza, and HIV was assessed through diagnostic ROC curve analysis. Results of the ROC analysis for DEGs are depicted in **Figure 4**, with the Area Under the Curve (AUC) graphs of the 22 DEGs in COVID-19, influenza, and HIV presented in **Figures 4A–D**, 4E-H, and 4I-L, respectively. As depicted in **Figure 4**, a limited number of DEGs exhibited an AUC of less than 0.7. Specifically, in COVID-19, these genes were *CD52*, *ISG15*, and *MMP9*; in influenza, they included *IFI44L*, *IFIT3*, *ISG15*, and *OAS1*; and in HIV, *OAS1* was identified. Excluding these genes, the AUC values for the diagnostic ROC curves corresponding to other differential genes in the three disease groups were consistently above 0.7. This suggests that these genes have the possibility to serve as biomarkers for the respective diseases.

### 3.3 Immune infiltration analysis in COVID-19, influenza,and HIV datasets

**Figure 5:**
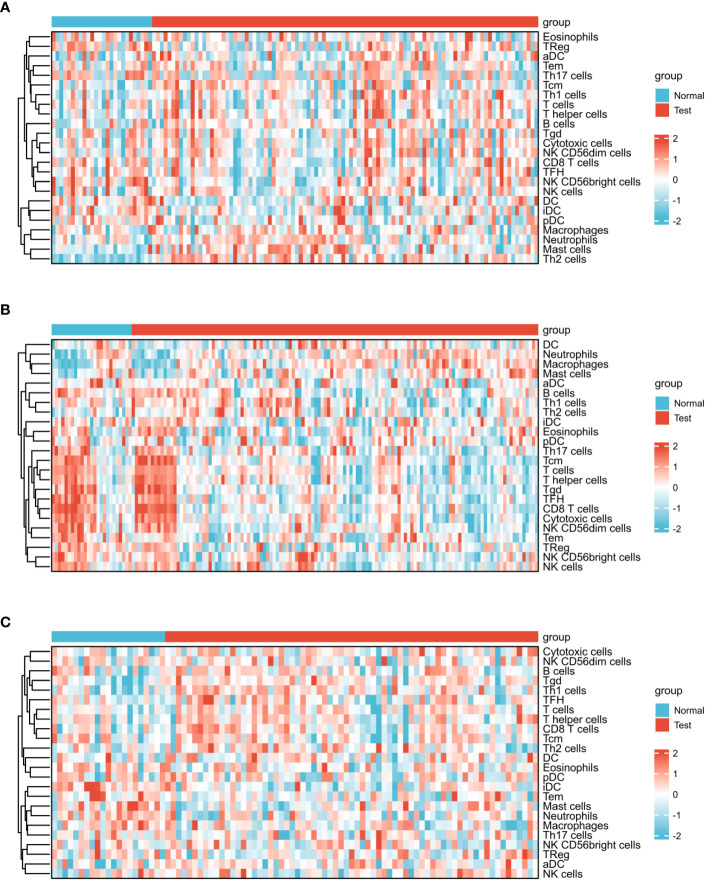
Expression analysis of infiltrated immune cells by ssGSEA algorithm among three diseases. **(A)** Heat map of COVID-19. **(B)** Heat map of Influenza. **(C)** Heat map of HIV.

**Figure 6:**
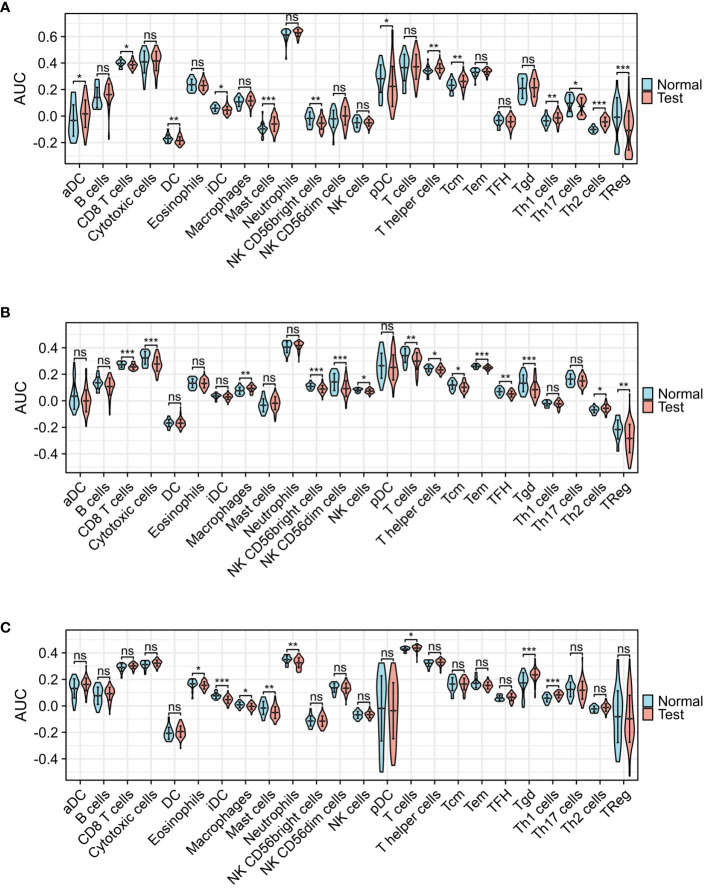
Group comparison graphs of infiltrated immune cells by ssGSEA algorithm among three diseases. **(A)** Infiltrated immune cells expression levels of COVID-19. **(B)** Infiltrated immune cells expression levels of Influenza. **(C)** Infiltrated immune cells expression levels of HIV. (*p<0.05, **p<0.01, ***p<0.001, ns meant no significant difference).

**Figure 7:**
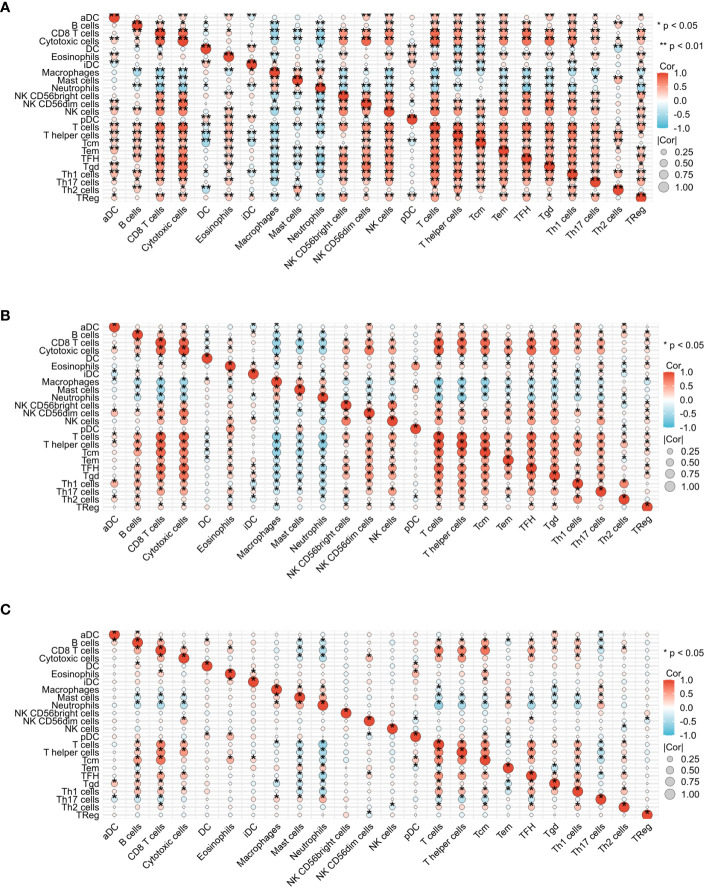
Correlation heat map analysis of infiltrated immune cells by ssGSEA algorithm among three diseases. **(A)** Correlation heat map of COVID-19. **(B)** Correlation heat map of Influenza. **(C)** Correlation heat map of HIV.

**Figure 5** presents the heat map results of immune infiltration analysis using the ssGSEA algorithm for three diseases. Specifically, **Figure 5A** illustrates the immune cell profile for COVID-19, **Figure 5B** for influenza, and **Figure 5C** for HIV, each representing 24 different immune cells. In **Figure 6**, the group comparison charts of immune infiltration analysis for COVID-19, influenza, and HIV reveal distinct expression levels for various immune cells between the disease and healthy control groups. In COVID-19 (**Figure 6A**), 13 immune cells, including aDC, CD8 T cells, DC, iDC, Mast cells, NK CD56 bright cells, pDC, T helper cells, Tcm, Th1 cells, Th17 cells, Th2 cells, and TReg, exhibited significant differences in expression. For influenza (**Figure 6B**), 14 immune cells, such as CD8 T cells, Cytotoxic cells, Macrophages, NK CD56bright cells, NK CD56dim cells, NK cells, T cells, T helper cells, Tcm, Tem, TFH, Tgd, Th2 cells, and TReg, displayed noticeable differences in expression. In HIV (**Figure 6C**), the expression levels of 8 immune cells, including Eosinophils, iDC, Macrophages, Mast cells, Neutrophils, T cells, Tgd, and Th1 cells, exhibited important differences between the disease and normal groups. **Figure 7** illustrates the correlation heat map of ssGSEA outcomes. **Figure 7A** displays the correlation heat maps of 24 immune cells in COVID-19, **Figure 7B** for influenza, and **Figure 7C** for HIV.

### 3.4 GO and KEGG analyses

**Figure 8:**
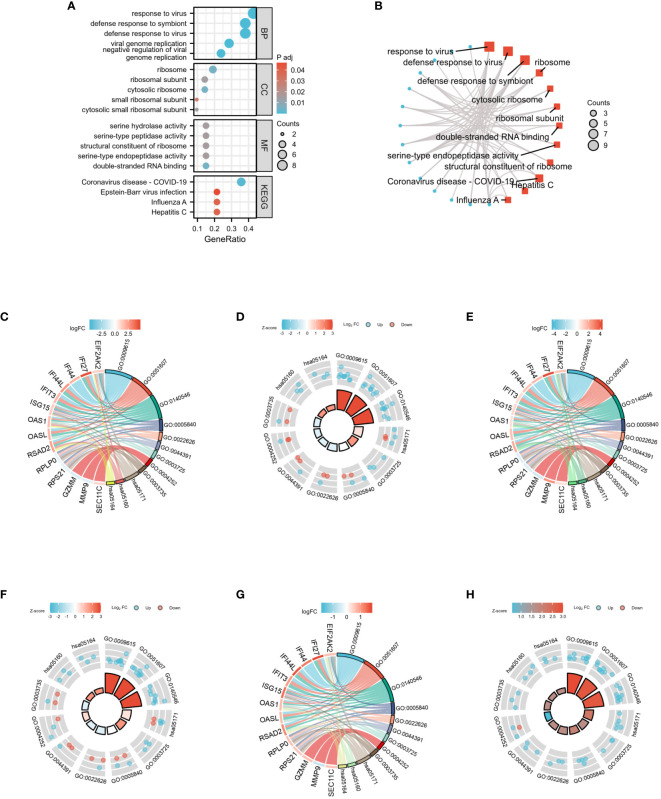
GO and KEGG functional enrichment analysis of 22 DEGs among three diseases. **(A)** The bubble graph of GO and KEGG functional enrichment analysis. **(B)** The network diagram of GO and KEGG functional enrichment analysis. **(C)** Chordal diagram of GO/KEGG-United logFC in COVID-19. **(D)** Loop graph of GO/KEGG-United logFC in COVID-19. **(E)** Chordal diagram of GO/KEGG-United logFC in Influenza. **(F)** Loop graph of GO/KEGG-United logFC in Influenza. **(G)** Chordal diagram of GO/KEGG-United logFC in HIV. **(H)** Loop graph of GO/KEGG-United logFC in HIV.

GO analysis was conducted by selecting the top 5 items from the BP, CC, and MF categories. Additionally, the top 4 KEGG were selected (**Figure 8A**). The DEGs exhibited significant enrichment, including the response to the virus of the BP category, high enrichment in ribosome activity of the CC category, and double-stranded RNA binding of the MF category. Moreover, this enrichment extended to the Coronavirus disease - COVID-19 pathway in the KEGG category, indicating its involvement in immunotherapy-related functional enrichment. To provide a more comprehensive explanation, the pathway enrichment analysis was visualized via bubble graph. The top three results from the BP, CC, MF, and KEGG categories were chosen to illustrate the enrichment analysis of specific biological pathways in the network diagram (**Figure 8B**). For the GO/KEGG-United logFC analysis in COVID-19, 22 DEGs were utilized, and chordal diagrams and loop graphs were generated, as shown in **Figures 8C, D**. Besides, the results of GOKEGG-United logFC analysis in Influenza are shown in **Figures 8E, F**. **Figures 8G, H** present the results of GOKEGG-United logFC analysis in HIV.

### 3.5 GSEA functional enrichment analysis of all genes among COVID-19, influenza, and HIV

**Figure 9:**
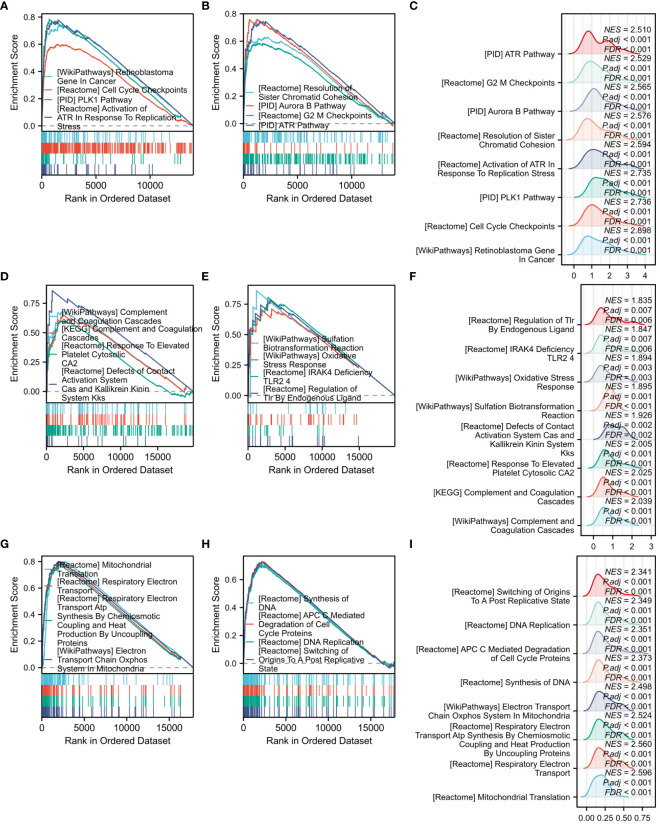
GSEA functional enrichment analysis of all genes among three diseases. **(A)** Classic graph of 1-4 pathways in COVID-19. **(B)** Classic graph of 5-8 pathways in COVID-19. **(C)** Mountain plot of 8 pathways in COVID-19. **(D)** Classic graph of 1-4 pathways in Influenza. **(E)** Classic graph of 5-8 pathways in Influenza. **(F)** Mountain plot of 8 pathways in Influenza. **(G)** Classic graph of 1-4 pathways in HIV. **(H)** Classic graph of 5-8 pathways in HIV. **(I)** Mountain plot of 8 pathways in HIV.

GSEA analysis was performed on all genes associated with COVID-19, yielding 8 significant pathways. These pathways encompassed the plk1 pathway, retinoblastoma gene in cancer, cell cycle checkpoints, resolution of sister chromatid cohesion, activation of atr in response to replication stress, aurora b pathway, G2 M checkpoints, and atr pathway (**Figures 9A, B**). **Figure 9C** presents the Mountain plot of these 8 pathways in COVID-19. Subsequently, GSEA analysis was carried out on all genes related to Influenza, revealing the 8 most significant pathways. These pathways included oxidative stress response, KEGG complement and coagulation cascades, WP complement and coagulation cascades, complement and coagulation cascades, response to elevated platelet cytosolic Ca2, sulfation biotransformation reaction, defects of contact activation system CAS and kallikrein-kinin system KKS, IRAK4 deficiency TLR2 4, and regulation of TLR by endogenous ligand (**Figures 9D, E**). A Mountain plot was generated to depict these 8 pathways in Influenza (**Figure 9F**). GSEA analysis of all genes in HIV was conducted, as shown in **Figures 9G, H**. Among the 8 identified pathways, the most significant were mitochondrial translation, respiratory electron transport, DNA synthesis, ATP synthesis by chemiosmotic coupling and heat production by uncoupling proteins, DNA replication, electron transport chain in the oxidative phosphorylation system of mitochondria, degradation of cell cycle proteins mediated by APC C, and switching of origins to a post-replicative state. **Figure 9I** displays the Mountain plot illustrating these 8 pathways in HIV.

### 3.6 PPIs and hub genes among COVID-19, influenza,and HIV

**Figure 10:**
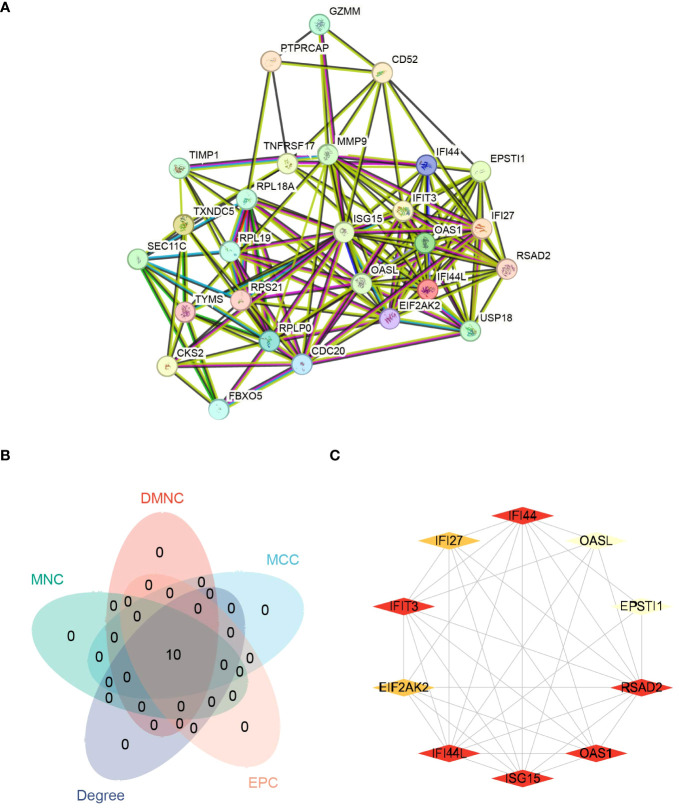
Protein-protein interaction networks (PPIs) and hub genes for common DEGs to COVID-19, Influenza and HIV. **(A)** Shared DEGs of COVID-19, Influenza and HIV in the PPIs (27 nodes,143 edges). **(B)** The Venn diagram of screened hub genes from MCC, DMNC, MNC, Degree and EPC 5 algorithms. **(C)** The red and yellow rhomboid nodes represent the top 10 hub genes and edges represent the interactions between nodes.

The interactions among the overlapping genes were explored utilizing the STRING database. The PPIs of shared DEGs comprised 27 nodes and 143 edges, as illustrated in **Figure 10A**. The identification of hub genes was achieved through PPIs analysis using a plugin for Cytoscape called Cytohubba. To determine the most influential top ten hub genes, we employed five algorithms—MCC, DMNC, Degree, MNC, and EPC. All five algorithms consistently identified the same top ten hub genes, as shown in **Figure 10B**. The MCC algorithm identified *FI44L*, *IFI44*, *RSAD2*, *ISG15*, *IFIT3*, *OAS1*, *EIF2AK2*, *IFI27*, *OASL*, and *EPSTI1* as the top 10 hub genes (**Figure 10C**). Hence, these identified hub genes could serve as effective biomarkers and contribute to advancing innovative therapeutic methods for these conditions.

### 3.7 The network of regulatory interactions of hub genes interconnected with miRNAs and TFs

**Figure 11:**
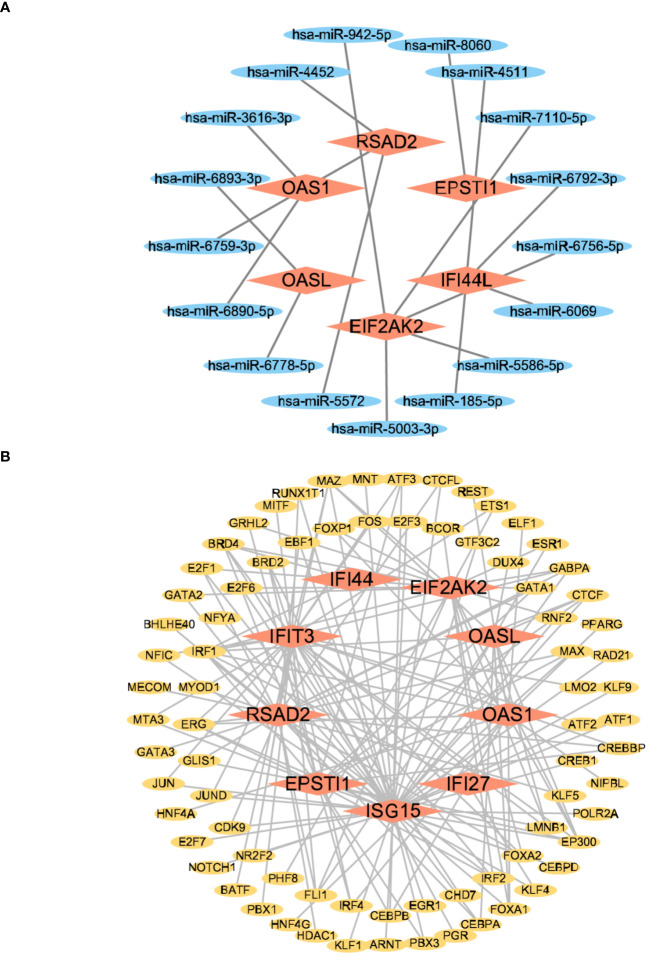
The interconnected regulatory interaction network of Hub genes–miRNAs and Hub genes–TFs. **(A)** Hub genes–miRNAs,red rhomboid nodes indicate Hub genes and blue oval nodes represent miRNAs (23 nodes,17 edges). **(B)** Hub genes–TFs,red rhomboid nodes indicate Hub genes and yellow oval nodes represent TFs (87 nodes,163 edges).

We conducted a screening process using the Mirwalk database, resulting in the identification of 499 Hub genes–miRNA interactions. Additionally, screening from the miRDB database yielded 699 Hub genes–miRNA interactions. The intersection of these databases revealed 17 Hub genes–miRNA interactions. Importantly, these interactions involved 6 hub genes—*IFI44L*, *RSAD2*, *OAS1*, *EIF2AK2*, *OASL*, and *EPSTI1*—along with 17 miRNAs in the interactive network. **Figure 11A** illustrates the intricate interactions between miRNA regulators and hub genes. The top 5 significant miRNAs identified in this network were hsa-miR-8060, hsa-miR-6890-5p, hsa-miR-5003-3p, hsa-miR-6893-3p, and hsa-miR-6069. Subsequently, we searched the hTFtarget database, identifying 295 Hub genes–TFs interactions. Further screening of the ChIP database resulted in 1338 Hub genes–TFs interactions. The intersection of these databases yielded 163 Hub genes–transcription factor interactions. These interactions were imported into Cytoscape, revealing 9 hub genes involved in this interactive network—*IFI44*, *RSAD2*, *ISG15*, *IFIT3*, *OAS1*, *EIF2AK2*, *IFI27*, *OASL*, and *EPSTI1*. **Figure 11B** displays 78 TFs due to the intersection between TFs and hub genes. The interactions between TFs and hub genes are depicted, identifying CREB1, CEBPB, EGR1, EP300, and IRF1 as the top five significant TFs.

### 3.8 The network of regulatory interactions between hub genes and chemicals, as well as hub genes and diseases

**Figure 12:**
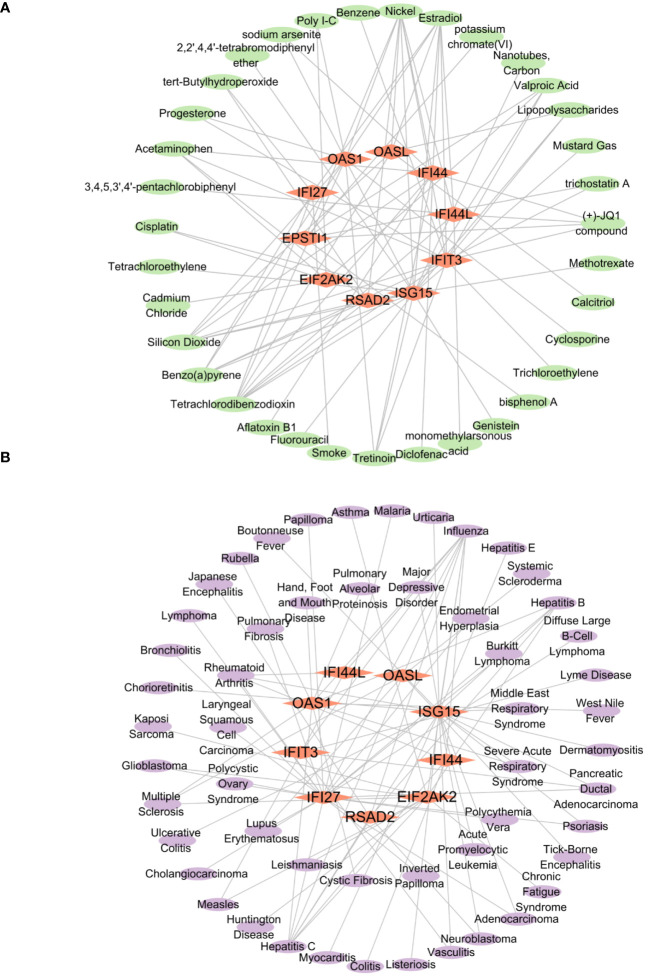
The interconnected regulatory interaction network of Hub genes–Drug chemicals and Hub genes–diseases. **(A)** Hub genes–Drug chemicals,red rhomboid nodes indicate Hub genes and green oval nodes represent Drug chemicals (45 nodes, 84 edges). **(B)** Hub genes–diseases,red rhomboid nodes indicate Hub genes and purple oval nodes represent diseases (62 nodes, 87 edges).

From the networkanalyst database, we identified 411 Hub genes–Drug Chemical interactions. Simultaneously, screening the Comparative Toxicogenomics Database produced 136 Hub genes–Drug Chemical interactions. The intersection of these two databases yielded 84 Hub genes–Drug Chemical interactions. Importing these interactions into Cytoscape revealed an interactive network of 10 hub genes and 35 Drug Chemicals. **Figure 12A** displays the top ten significant drug chemicals, including estradiol, progesterone, tretinoin, calcitriol, fluorouracil, methotrexate, lipopolysaccharide, valproic acid, silicon dioxide, and cyclosporine. These potential drugs could act as medicinal targets and interventions for COVID-19, Influenza, and HIV. Following a similar approach, we screened the DisGeNET database, identifying 888 Hub genes–disease names. Subsequently, screening the MalaCards database resulted in 2084 Hub genes–disease names. The intersection of these two databases yielded 87 Hub genes–disease names, which were imported into Cytoscape and displayed. The interaction network revealed the involvement of 9 hub genes and 53 diseases. The examination of gene-disease correlations highlighted that influenza, asthma, major depressive disorder, lymphoma, glioblastoma, cholangiocarcinoma, pancreatic ductal adenocarcinoma, acute promyelocytic leukemia, hepatitis C, and hepatitis B showed the highest level of coordination with the reported hub genes. These findings suggest that COVID-19, influenza, and HIV share common characteristics with these diseases. **Figure 12B** displays the connection between genes and diseases.

### 3.9 Prediction of hub genes-RBPs and qPCR verification

**Figure 13:**
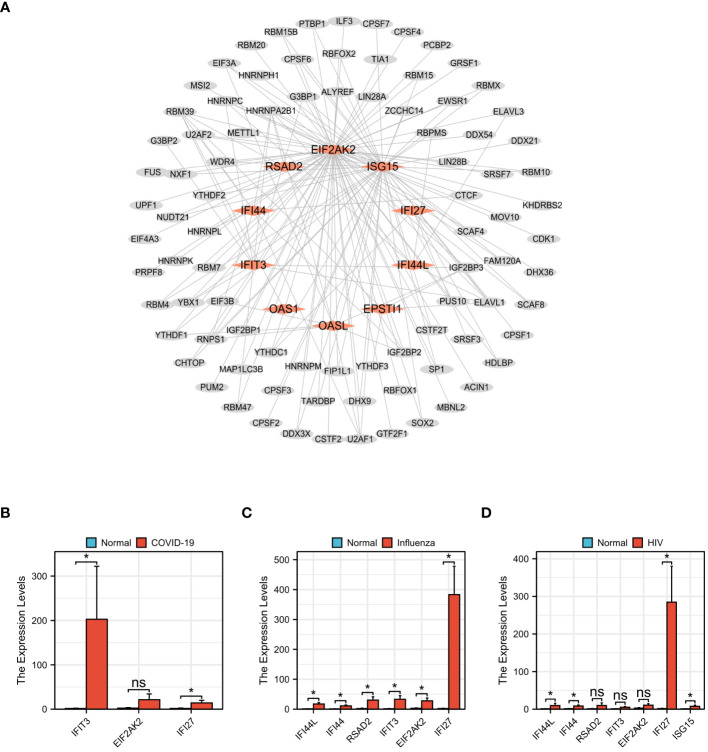
Prediction of Hub genes–RBPs and qPCR validation among COVID-19, Influenza and HIV. **(A)** Hub genes–RBPs,red rhomboid nodes indicate Hub genes and gray oval nodes represent RBPs (98 nodes, 164 edges). **(B)** The mRNA expression levels of IFIT3,EIF2AK2,IFI27 in COVID-19. **(C)** The mRNA expression levels of IFI44L,IFI44,RSAD2,IFIT3,EIF2AK2,IFI27 in Influenza. **(D)** The mRNA expression levels of IFI44L,IFI44,RSAD2,IFIT3,EIF2AK2,IFI27,ISG15 in HIV. (*p<0.05, ns meant no significant difference).

We retrieved 164 hub genes–RBPs from the starBase database and inputted them into Cytoscape. The resulting interaction network revealed the involvement of 10 hub genes and 88 RBPs, suggesting a shared characteristic between these RBPs and COVID-19, Influenza, and HIV. The hub genes–RBPs association is depicted in **Figure 13A**. The top five most important RBPs identified were RBM39, U2AF1, ELAVL1, IGF2BP2, and HNRNPA2B1. In the gene expression validation experiment for the COVID-19 group, significant differences were viewed between the disease and normal groups for the *IFIT3* and *IFI27* genes, while no notable distinction was found in the *EIF2AK2* gene (**Figure 13B**). The influenza group’s validation experiment showed significant differences in all six genes (*IFI44L*, *IFI44*, *RSAD2*, *IFIT3*, *EIF2AK2*, and *IFI27*) (**Figure 13C**). In the HIV group, four genes (*IFI44L*, *IFI44*, *IFI27*, and *ISG15*) exhibited significant differences, while the remaining three genes (*RSAD2*, *IFIT3*, and *EIF2AK2*) did not (**Figure 13D**).

### 3.10 Complex interrelationships of hub gene, miRNA, transcription factor, drug chemical, disease, and RBP

**Figure 14:**
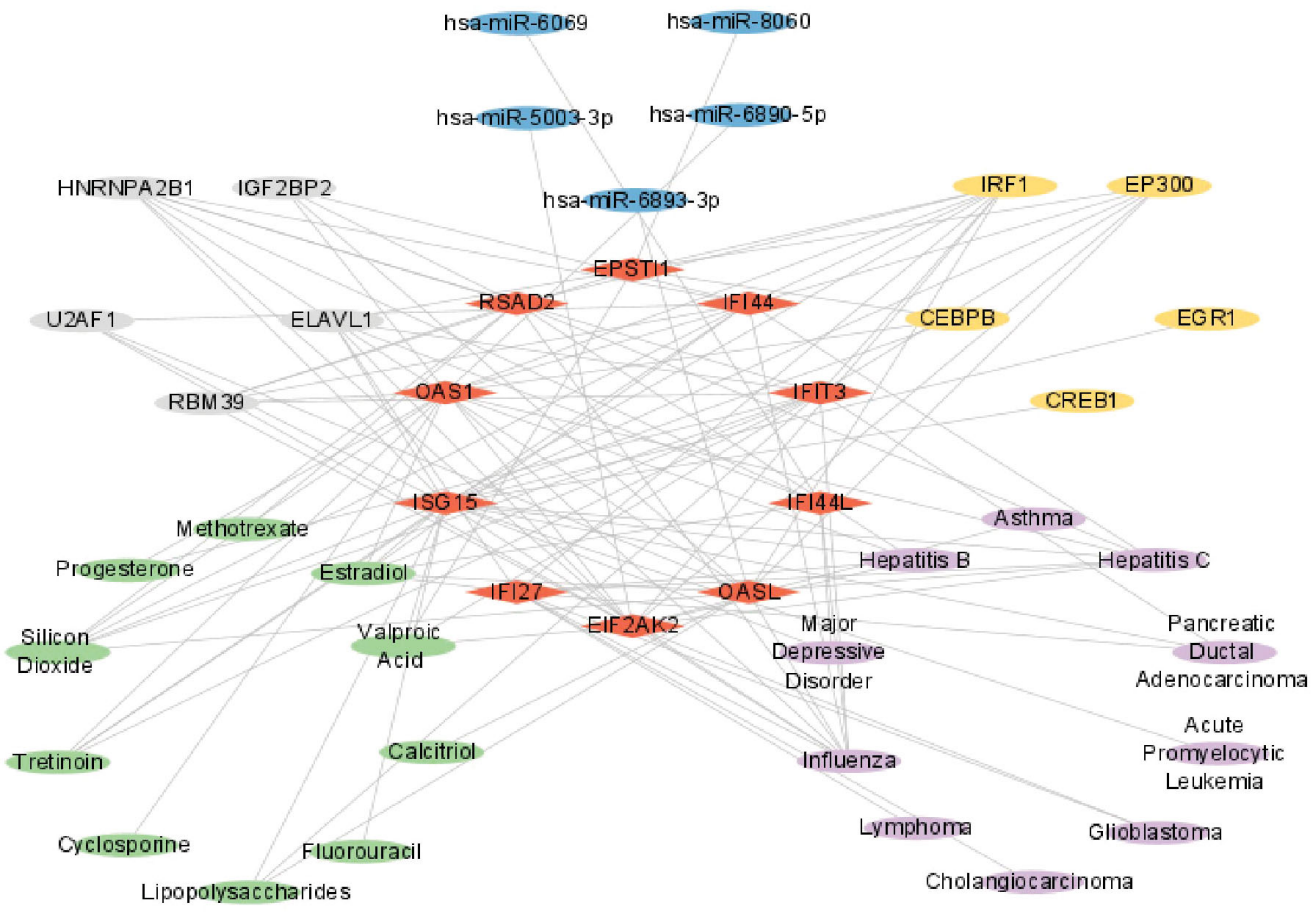
Complex interrelationships of Hub gene, miRNA, transcription factor, drug chemical, disease, and RBP. Red rhomboid nodes indicate Hub genes, blue oval nodes represent miRNAs, yellow oval nodes represent transcription factors, green oval nodes represent Drug chemicals, purple oval nodes represent diseases, and gray oval nodes represent RBPs(45 nodes, 114 edges).

In summary, these six molecules exhibited complex interrelationships. For instance, hsa-miR-6890-5p, CEBPB, Cyclosporine, Influenza, and RBM39 have interactive relationships centered around OAS1. Similarly, hsa-miR-5003-3p, EP300, Valproic Acid, Influenza, and ELAVL1 have interactive relationships centered around EIF2AK2, while hsa-miR-6893-3p, EP300, Calcitriol, Influenza, and HNRNPA2B1 exhibit interactive relationships centered around OASL. **Figure 14** illustrated a network diagram that highlights the mutual regulatory relationships among the six distinct types of molecules mentioned above. The six distinct types of molecules complex interrelationships table can be found in **Supplementary Table 4**.

## 4 Discussion

A notable increase in studies revealing potential connections between various diseases has been observed in recent years. Consequently, exploring the correlations among different illnesses represents a promising avenue for future research (32–34). COVID-19 and influenza, both highly contagious respiratory disorders, manifest comparable clinical symptoms due to shared pathogenicity and gene expression regulation in the host body (35, 36). HIV belongs to the Retroviridae family and the Lentivirus genus (37). It significantly impacts the well-being of individuals, families, communities, and the economic and social welfare of nations. Recent research suggests that individuals living with HIV have an increased susceptibility to contracting SARS-CoV-2 and are more likely to experience fatal outcomes from COVID-19 compared to those without HIV (38–40).

To research the molecular mechanisms underlying the worsened prognosis of HIV patients upon simultaneous infection with COVID-19 and influenza and to understand the regulatory role of these three viruses in host gene expression, we acquired sequencing data for COVID-19 (GSE157103), influenza (GSE185576), and HIV (GSE195434). Following this, we conducted a differential analysis of the three datasets. Among the 22 DEGs, it is noteworthy that only three genes, namely *ISG15*, *MMP9*, and *OAS1*, did not exhibit statistically significant differences between the disease group and the healthy control group across all three datasets. This finding could be attributed to several factors including the limitations of sample size, individual variations, and experimental design. This observation demonstrates the complexity and variability of differentially expressed genes in different infection states and requires further study and verification.

By intersecting the resulting DEGs from these three analyses, we further scrutinized the common DEGs, leading to the identification of 10 hub genes: *IFI44L*, *IFI44*, *RSAD2*, *ISG15*, *IFIT3*, *OAS1*, *EIF2AK2*, *IFI27*, *OASL*, and *EPSTI1*. These genes were significantly up-regulated in COVID-19, exerting roles in immune regulation. Previous research has highlighted the up-regulation of genes such as *IFI44L*, *IFFI44*, *RSAD2*, *OAS1*, *EPSTI1*, and *OSAL* in COVID-19, contributing to immune regulation (41). Notably, during the treatment of COVID-19, the down-regulation of genes like *IFI27*, which possesses antiviral effects and activates neutrophils, aligns with the increased inflammatory response observed in these patients (42). In individuals affected by influenza virus infection, *IFFI44L*, *ISG15*, *IFIT3*, and *RSAD2* are crucial antiviral factors inhibiting infection within alveolar basal epithelial cells (43). Analysis of kinases, phosphatases, and related signaling factors also reveals heightened activity of *EIF2AK2* in patients with HIV-associated dementia compared to those with mild neurocognitive disorder. Thus, *EIF2AK2* may play a significant role in HIV-1-associated neuropathogenesis in HIV-infected individuals (44).

To enhance our comprehension of how HIV-induced immunocompromise influences the acquisition and clearance of infection and elucidate shared mechanisms among COVID-19, influenza, and HIV, we conducted GO and KEGG analyses. Additionally, we employed the Xiantao tool to perform GO analysis on BP, CC, and MF associated with the shared DEGs related to these three diseases. The significantly enriched biological processes of these shared DEGs were related to responding to viruses, defense responses against viruses, defense response to symbionts, negative regulation of viral genome replication, and viral genome replication.

Based on Kalil and colleagues’ research, the progression of influenza virus infection entails an inflammatory process within the respiratory tract. This process is initiated when the virus directly infects the cells lining the respiratory system, immune responses, both innate and adaptive, are triggered. The primary objective of these responses is to effectively manage contagious diseases (45). Concerning the significantly enriched Cellular Components of these common DEGs, there is a notable concentration in ribosome-related terms, including ribosome, ribosomal subunit, cytosolic ribosome, small ribosomal subunit, and cytosolic small ribosomal subunit. Perturbations in ribosome production are associated with conditions such as cancer, developmental disorders, and viral infections, emphasizing the pivotal role of ribosomes in biological processes (46). Significantly enriched GO terms of these shared DEGs were also associated molecular functions such as binding double-stranded RNA, serine-type endopeptidase activity, the structural component of ribosomes, serine-type peptidase activity, and serine hydrolase activity. This, underscores the crucial reliance of viruses on ribosomes for synthesizing polypeptides and generating polypeptide molecules (47, 48).

From a perspective of gene expression regulation, we identified 22 shared genes across the datasets of COVID-19, influenza, and HIV. The top 4 KEGG pathways enriched in these genes were related to COVID-19, Hepatitis C, Influenza A, and Epstein-Barr virus infection, emphasizing distinctions between SARS-CoV-2 and influenza virus infections (49). Enhancing our understanding of how HIV infection contributes to increased mortality risk in COVID-19 and influenza involves improving the identification of crucial gene ontology and molecular pathways.

GSEA analysis of all genes in the three datasets revealed specific pathway enrichments. The COVID-19 dataset showed enrichment in the PLK1 pathway, associated with stem cell cancer (50), glioma (51), and lung adenocarcinoma (52). The influenza dataset exhibited enrichment in the oxidative stress response signaling pathway, a potential therapeutic target for gastric cancer (53). Furthermore, the HIV dataset showed enrichment in the mitochondrial translation pathway, which is crucial for the continuous cytotoxicity of T lymphocytes (CTL) (54).

Next, we assessed the accumulation of immune cells in tissues or lesions and found a notable decrease in the abundance of CD8 T cells, bright CD56 cells of natural killer, and regulatory T cells among individuals with COVID-19 and influenza. Additionally, infections caused by COVID-19 and HIV in patients exhibited a decrease in the number of immature dendritic cells, indicating a depletion of immune cells and compromised immune function (21). In addition, during the H1N1 epidemic, about half of influenza patients experienced a reduced CD4:CD8 ratio (55).

Notably, patients with COVID-19 and HIV exhibited an increase in Th1 cells. Th1 lymphocytes play a pivotal role in the immune system by activating cellular immunity, fostering inflammatory responses, enhancing cytotoxicity, and promoting antibody production. They contribute significantly to combating infections, regulating tumor development, and maintaining immune system balance. A notable upregulation of immune cells and inflammatory markers was observed in all three diseases. While these findings may have been reported in previous studies, we believed that these findings still hold significant implications. Firstly, the identification of specific immune cell types has the potential to impact disease progression and treatment in the context of the three specific diseases examined. Secondly, there is a need for further exploration of immune cell subtypes within these diseases and their respective roles in pathological processes. Additionally, the upregulation of inflammatory markers may be associated with distinct clinical characteristics of patients, warranting further investigation. Moreover, our future investigations will focus on examining the interactions between these immune cells and other biomarkers to gain a comprehensive understanding. The presence and proper functioning of immune cells are essential for a impactful immune response, involving monitoring potential threats, controlling inflammation, and assessing prognosis. A more profound comprehension of the characteristics and mechanisms of immune infiltration holds valuable insights for diagnosing, treating, and preventing diseases.

MiRNAs, by binding to the mRNA of target genes and influencing their translation or degradation, play a pivotal role in controlling gene expression levels. We utilized two well-established and validated databases, namely miRWalk and miRDB, to investigate the relationship between miRNAs and mRNAs. These databases contain a vast collection of experimentally verified interactions between miRNAs and their target mRNAs. Based on these data, various algorithms such as seed matching, conservation analysis, RNA secondary structure prediction, and machine learning models are used to predict potential miRNAs that are likely associated with the target mRNA. This integrated approach allowed us to comprehensively explore and identify putative miRNA-mRNA interactions with high confidence.

Studies suggest that hsa-miR-8060 could be a biomarker for diagnosing H1N1 virus infectious diseases (56). Hsa-miR-6890-5p regulates dehydrogenase/reductase 9 (DHRS9), a significant biomarker for atherosclerosis (57). Hsa-miR-5003-3p exhibits a correlation with both ICU admission and blood endophenotypes, thereby exerting regulatory influence over the host response to COVID-19 (58). Hsa-miR-6893-3p is a promising miRNA for SARS-CoV-2 infection, targeting the ORF1ab and spike protein of the SARS-CoV-2 virus (59). Hsa-miR-6069, an up-regulated predicted gene, represents a potentially crucial candidate for molecular targets and diagnostic biomarkers for COVID-19 (60). The analysis of predicted miRNAs from mRNA reveals the intricate interactions between miRNAs and their target genes. It further determines the specific set of genes that may be regulated by miRNAs, allowing for the identification of potential regulatory pathways. Moreover, it uncovers disease-associated miRNAs and their target genes, thereby predicting gene-centric, potential therapeutic targets. This comprehensive analysis provides pivotal insights into miRNA regulation and gene expression control, serving as a theoretical foundation for the development of novel treatment strategies and therapeutics.

Transcription factors, a category of proteins, play a significant role in regulating gene transcription by binding to DNA. We employed two widely recognized databases, namely hTFtarget and ChIPBase, to investigate the transcription factor binding site information. These databases provide a comprehensive collection of experimentally validated transcription factor binding site data. Through a comparative analysis of the target mRNA sequence with the binding site patterns documented in these databases, we successfully predicted the potential transcription factors that are likely to bind to the target mRNA and regulate its expression. This approach allowed us to gain insights into the regulatory mechanisms underlying the target mRNA and provided valuable information regarding the involvement of specific transcription factors in its regulation.

CREB1, a central transcription factor in the network, is linked to changes in protein expression induced by the SARS-CoV-2 spike S1 (61). CEBPB, identified in the differential analysis of bronchoalveolar lavage fluid from SARS-CoV-2-positive individuals, is involved in chemokine signaling and immunometabolism (62). EGR1, a versatile transcription factor found in mammals, has been associated with various viral infections, including HSV-1, HIV, and EBV (63). EP300, a crucial target of glycosides, impacts viral myocarditis, chemotaxis of monocytes and macrophages, and T-cell activation, leading to antibody responses, potentially serving as a therapeutic target for influenza A viruses (64). MicroRNA-132-3p reportedly hinders the type I IFN response and facilitates infection of the H1N1 influenza A virus by targeting IRF1 (65). Transcription factors play a significant role in regulating normal physiological processes, development, and the occurrence of diseases.

We utilized two established databases, namely networkanalyst and Comparative Toxicogenomics Database, to explore the relationship between drug and target genes. These databases contain comprehensive records of interactions between drug and their target genes. By comparing the association of the target mRNA with known drug target genes, we were able to predict potential drug chemicals that may interact with the target mRNA and hold therapeutic potential. Currently, drugs like molnupiravir, azivudine, paxlovid, and remdesivir have been approved for COVID-19 treatment. However, there is currently no evidence indicating that specific antiretroviral treatments either enhance or worsen therapeutic effects for infections caused by COVID-19 and HIV in patients or can prevent SARS-CoV-2 infection (66). Hence, the development of a safe and efficacious pharmaceutical intervention for the treatment of AIDS patients co-infected with COVID-19 and influenza is of utmost importance. This study has identified various drug chemicals, including estradiol, progesterone, tretinoin, calcitriol, fluorouracil, methotrexate, lipopolysaccharide, valproic acid, silicon dioxide, and cyclosporine, with the potential to simultaneously treat COVID-19, influenza, and HIV. Research has indicated the participation of estradiol in regulating HIV infection (67), and it has a substantial effect on reducing COVID-19 mortality (68). Calcitriol has recently discovered as a potentially efficacious therapeutic agent for the management of COVID-19 (69).

Retinoic acid can reportedly enhance the function and development of epithelial and endothelial barriers, inhibiting inflammation-related barrier damage through modulation of immune cell activity (70). Callaghan et al. conducted research supporting these findings, revealing that the breakdown of retinoic acid is hindered in individuals with COVID-19 sepsis, and adding retinoic acid could offer a fresh approach to treating COVID-19 (71). In the context of a Syrian hamster model, the investigation revealed that progesterone exhibits the potential to alleviate the severity of COVID-19 pneumonia (72). The combination of 5-Fluorouracil with deoxyribonucleosides and deoxyribose exhibits promise as a prospective therapeutic strategy for the treatment of coronavirus infections (73). Additionally, Methotrexate is recognized as a safe immunosuppressant for use during the COVID-19 pandemic (74).Through the analysis of predicted drug chemicals from RNA, a comprehensive understanding of the intricate interactions and regulatory mechanisms between drug candidates and disease-related genes can be achieved. This analysis serves as a solid theoretical foundation and offers valuable guidance for the discovery of novel treatment strategies, the development of personalized therapeutic approaches, and the evaluation of the potential efficacy of drugs.

We employed two widely accessible databases, namely DisGeNET and MalaCards, which provide curated collections of disease-related genomics datasets. These databases encompass comprehensive lists of genes associated with various diseases. Through a comparative analysis and correlation of the target mRNA with these known disease-associated genes, we were able to infer potential connections between the target mRNA and specific diseases. This approach enabled us to gain insights into the potential involvement of the target mRNA in disease pathogenesis and provided valuable information for further investigations into disease mechanisms and therapeutic interventions.

Genetic disease analysis revealed that hub genes are associated with a range of illnesses in COVID-19, influenza, and HIV, encompassing influenza, asthma, major depressive disorder, lymphoma, glioblastoma, cholangiocarcinoma, pancreatic ductal adenocarcinoma, acute promyelocytic leukemia, hepatitis C, and hepatitis B. Respiratory viruses like influenza can frequently trigger acute asthma episodes (75, 76). COVID-19 poses a significant threat to individuals with mental disorders, especially depression and schizophrenia (77). Numerous studies demonstrate a significant correlation between COVID-19 and various cancers, such as breast, colon, stomach, and prostate tumors (78). Considering the gravity of cancer and the compromised immune system, individuals diagnosed with COVID-19 face an increased likelihood of mortality (79). According to a study, pre-existing chronic liver disease was observed in approximately 2-11% of patients diagnosed with COVID-19 (80).

RNA-binding proteins represent ubiquitous proteins found in various cellular contexts and play a key role in regulating post-transcriptional processing, translation, stability, transport, and modification of RNA. We utilized the ENCORI database, a publicly available resource that provides comprehensive information on RNA binding protein (RBP) binding sites. This database contains curated records of experimentally verified RBP binding site data. Through a comparative analysis of the target mRNA sequence with the binding site patterns documented in the ENCORI database, we were able to predict the RBPs that are likely to bind to the target mRNA and exert regulatory control over its expression. In terms of mechanism, arginine alters the metabolism of liver cancer by attaching to RNA-binding protein 39 (RBM39) to regulate the expression of metabolic genes (81). U2AF1 mutations are more common in younger patients with myelodysplastic syndromes (MDS) and, despite remaining stable throughout clinical progression, are linked to a poorer prognosis. This mutation exhibits the potential to function as a biomarker for risk stratification (82). The analysis of ELAVL1 expression in distinct respiratory cell populations among individuals with COVID-19 and COPD unveiled a noteworthy positive correlation between ELAVL1 and ACE2, particularly in cells affected by COPD (83). In the context of the Women’s Interagency HIV Study (WIHS) research, the identification of IGF2BP2 as a significant determinant in antiretroviral therapy has revealed its capacity to modulate the genetic impacts of established risk variants linked to type 2 diabetes (84). The ribonucleoprotein hnRNPA2B1 represents a potential target for treating recurrent thymic epithelial tumors (85). By interacting with RNA molecules, RBP actively participates in the intricate regulatory network of gene expression in cells, playing a vital role in regulating normal cellular functions and adaptive responses.

In this study, we employed an integrated approach to analyze multiple datasets, and the results were interconnected by identifying common key nodes represented by hub genes. Among the identified hub genes, there were shared regulatory genes involved in miRNA prediction, transcription factor prediction, drug chemical prediction, disease prediction, and RBP prediction. The regulatory relationships between each pair of these molecules are as follows: 1.miRNA-mediated regulation: miRNA binds to the 3’ untranslated region (3’ UTR) or other regulatory regions of mRNA, leading to mRNA degradation or inhibition of its translation process. 2.Transcription factor-mediated regulation: Transcription factors can bind to the promoter or enhancer region of mRNA, directly influencing the transcriptional process of mRNA. 3.Drug chemical-mediated regulation: Drug compounds interact with specific targets on mRNA, affecting mRNA stability, degradation, or translation processes. 4.Disease-related regulation: Abnormal expression or mutation of mRNA can serve as disease markers and influence disease progression and clinical manifestations. 5.RNA-binding protein (RBP)-mediated regulation: RBPs interact with specific regions of mRNA, influencing mRNA stability, post-transcriptional modification, transport, and other processes. Additionally, miRNAs can regulate the expression levels of transcription factors by binding to the mRNA of transcription factors. These interrelationships are crucial for understanding cellular regulation, disease mechanisms, and the development of therapeutic interventions. A deeper exploration of these relationships can provide novel perspectives and strategies for disease prevention, diagnosis, and treatment.

To sum up, our research presents several strengths. We utilized blood samples from the GEO database containing COVID-19, influenza, and HIV, identifying central genes that could significantly contribute to the progression of these three illnesses. Moreover, we uncovered connections among these diseases, providing new perspectives into the molecular processes of concurrent getting sick with COVID-19 and influenza viruses in individuals with HIV. Additionally, we identified 10 drug chemicals that could support as potential therapeutic drug candidates for managing patients with COVID-19, influenza, and HIV.

Nevertheless, despite the strengths of our research, it is crucial to acknowledge certain limitations. Firstly, we obtained study data from the publicly available GEO database and performed gene expression validation on a limited number of samples. Secondly, there is a lack of comparable patient demographics and matching confounding variables. We will further collect data or strengthen collaborative research in subsequent studies to obtain more comprehensive and comparable patient information. Thirdly, the study currently lacks a comprehensive analysis of co-infections involving all three diseases. We will actively collect and analyze co-infection data to validate the results obtained from single infection data in this study. Additionally, it is essential to validate the biological roles of central genes and assess the efficacy and safety of potential medications through either fundamental or clinical experiments. Additional investigation is necessary to delve into the molecular mechanisms underlying COVID-19, influenza, and HIV.

## 5 Conclusion

The current investigation offers a comprehensive analysis of shared molecular targets, signaling pathways, drug chemicals, and potential biomarkers associated with COVID-19, influenza, and HIV. These discoveries hold the potential to enhance the accuracy of diagnosing and treating individuals with HIV who are also infected with COVID-19 and influenza.

## Data availability statement

The original contributions presented in the study are included in the article/**Supplementary Material**. Further inquiries can be directed to the corresponding author.

## Ethics statement

The studies involving humans were approved by The Medical Research Ethics Committee at the First Affiliated Hospital of Jinzhou Medical University. The studies were conducted in accordance with the local legislation and institutional requirements. The participants provided their written informed consent to participate in this study. Written informed consent was obtained from the individual(s) for the publication of any potentially identifiable images or data included in this article.

## Author contributions

ZZ: Data curation, Methodology, Validation, Visualization, Writing – original draft. HJ: Data curation, Funding acquisition, Methodology, Resources, Writing – original draft. XZ: Data curation, Funding acquisition, Methodology, Resources, Writing – original draft. MB: Validation, Visualization, Writing – review & editing. KZ: Validation, Visualization, Writing – review & editing. JT: Funding acquisition, Resources, Writing – review & editing. BD: Validation, Visualization, Writing – review & editing. LM: Validation, Visualization, Writing – review & editing. PQ: Validation, Visualization, Writing – review & editing. BH: Funding acquisition, Project administration, Resources, Supervision, Writing – review & editing.

## References

[1] Lu R Zhao X Li J Niu P Yang B Wu H. Genomic characterisation and epidemiology of 2019 novel Coronavirus: implications for virus origins and receptor binding. Lancet (London England). (2020) 395:565–74. doi: 10.1016/s0140-6736(20)30251-8 PMC7159086 32007145

[2] Finkel Y Mizrahi O Nachshon A Weingarten-Gabbay S Morgenstern D Yahalom-Ronen Y. The coding capacity of Sars-Cov-2. Nature. (2021) 589:125–30. doi: 10.1038/s41586-020-2739-1 32906143

[3] Cooper TJ Woodward BL Alom S Harky A. Coronavirus disease 2019 (Covid-19) outcomes in Hiv/Aids patients: A systematic review. HIV Med. (2020) 21:567–77. doi: 10.1111/hiv.12911 PMC7405326 32671970

[4] Zhu N Zhang D Wang W Li X Yang B Song J. A novel Coronavirus from patients with pneumonia in China, 2019. N Engl J Med. (2020) 382:727–33. doi: 10.1056/NEJMoa2001017 PMC7092803 31978945

[5] Abd El-Aziz TM Stockand JD. Recent progress and challenges in drug development against Covid-19 Coronavirus (Sars-Cov-2) - an update on the status. Infect Genet Evol. (2020) 83:104327. doi: 10.1016/j.meegid.2020.104327 32320825 PMC7166307

[6] Calabrese LH Naides SJ. Viral arthritis. Infect Dis Clin North Am. (2005) 19:963–80. doi: 10.1016/j.idc.2005.09.002 16297742

[7] Franssila R Hedman K. Infection and musculoskeletal conditions: viral causes of arthritis. Best Pract Res Clin Rheumatol. (2006) 20:1139–57. doi: 10.1016/j.berh.2006.08.007 17127201

[8] Hu B Guo H Zhou P Shi ZL. Characteristics of Sars-Cov-2 and Covid-19. Nat Rev Microbiol. (2021) 19:141–54. doi: 10.1038/s41579-020-00459-7 PMC7537588 33024307

[9] Sohrabi C Alsafi Z O'Neill N Khan M Kerwan A Al-Jabir A. World health organization declares global emergency: A review of the 2019 novel Coronavirus (Covid-19). Int J Surg. (2020) 76:71–6. doi: 10.1016/j.ijsu.2020.02.034 PMC7105032 32112977

[10] Ryabkova VA Churilov LP Shoenfeld Y. Influenza infection, Sars, Mers and Covid-19: cytokine storm - the common denominator and the lessons to be learned. Clin Immunol. (2021) 223:108652. doi: 10.1016/j.clim.2020.108652 33333256 PMC7832378

[11] Chen N Zhang B Deng L Liang B Ping J. Virus-host interaction networks as new antiviral drug targets for Iav and Sars-Cov-2. Emerg Microbes Infect. (2022) 11:1371–89. doi: 10.1080/22221751.2022.2071175 PMC9132403 35476817

[12] Lasry A Medley A Behel S Mujawar MI Cain M Diekman ST. Scaling up Testing for Human Immunodeficiency Virus Infection among Contacts of Index Patients - 20 Countries, 2016-2018. MMWR Morb Mortal Wkly Rep. (2019) 68:474–7. doi: 10.15585/mmwr.mm6821a2 PMC6542477 31145718

[13] Jordan RE Adab P Cheng KK. Covid-19: risk factors for severe disease and death. BMJ. (2020) 368:m1198. doi: 10.1136/bmj.m1198 32217618

[14] Siedner MJ Triant V. Undetectable = Untransmittable and your health: the personal benefits of early and continuous therapy for Hiv infection. J Infect Dis. (2019) 219:173–6. doi: 10.1093/infdis/jiy445 PMC6306014 30032272

[15] Tesoriero JM Swain CE Pierce JL Zamboni L Wu M Holtgrave DR. Covid-19 Outcomes among Persons Living with or without Diagnosed Hiv Infection in New York State. JAMA Netw Open. (2021) 4:e2037069. doi: 10.1001/jamanetworkopen.2020.37069 33533933 PMC7859843

[16] Andrew B Mary-Ann D Hannah H Muzzammil I Erna M Vundle Z. Risk factors for Coronavirus disease 2019 (COVID-19) death in a population cohort Study from the Western Cape Province, South Africa. Clin Infect Dis. (2021) 73:e2005-e2015. doi: 10.1093/cid/ciaa1198 32860699 PMC7499501

[17] Dong Y Li Z Ding S Liu S Tang Z Jia L. Hiv infection and risk of Covid-19 mortality: A meta-analysis. Med (Baltimore). (2021) 100:e26573. doi: 10.1097/MD.0000000000026573 PMC8257842 34190201

[18] Cohen C Moyes J Tempia S Groome M Walaza S Pretorius M. Mortality amongst patients with influenza-associated severe acute respiratory illness, South Africa, 2009-2013. PLoS One. (2015) 10:e0118884. doi: 10.1371/journal.pone.0118884 25786103 PMC4365037

[19] Gonzalez Alvarez DA Lopez Cortes LF Cordero E. Impact of Hiv on the severity of influenza. Expert Rev Respir Med. (2016) 10:463–72. doi: 10.1586/17476348.2016.1157474 26918376

[20] Piroth L Cottenet J Mariet AS Bonniaud P Blot M Tubert-Bitter P. Comparison of the characteristics, morbidity, and mortality of Covid-19 and seasonal influenza: A nationwide, population-based retrospective cohort study. Lancet Respir Med. (2021) 9:251–9. doi: 10.1016/S2213-2600(20)30527-0 PMC7832247 33341155

[21] Bai Y Tao X. Comparison of Covid-19 and influenza characteristics. J Zhejiang Univ Sci B. (2021) 22:87–98. doi: 10.1631/jzus.B2000479 33615750 PMC7885750

[22] Talbot HK Martin ET Gaglani M Middleton DB Ghamande S Silveira FP. Coronavirus disease 2019 (Covid-19) versus influenza in hospitalized adult patients in the United States: differences in demographic and severity indicators. Clin Infect Dis. (2021) 73:2240–7. doi: 10.1093/cid/ciab123 PMC8195096 34050659

[23] Ye Z Wang Y Colunga-Lozano LE Prasad M Tangamornsuksan W Rochwerg B. Efficacy and safety of corticosteroids in Covid-19 based on evidence for Covid-19, other Coronavirus infections, influenza, community-acquired pneumonia and acute respiratory distress syndrome: A systematic review and meta-analysis. CMAJ. (2020) 192:E756–E67. doi: 10.1503/cmaj.200645 PMC7828900 32409522

[24] Giacomelli A Pezzati L Conti F Bernacchia D Siano M Oreni L. Self-reported olfactory and taste disorders in patients with severe acute respiratory Coronavirus 2 infection: A cross-sectional study. Clin Infect Dis. (2020) 71:889–90. doi: 10.1093/cid/ciaa330 PMC7184514 32215618

[25] Blake JA Christie KR Dolan ME Drabkin HJ Hill DP Ni L. Gene ontology consortium: going forward. Nucleic Acids Res. (2015) 43:D1049–56. doi: 10.1093/nar/gku1179 PMC4383973 25428369

[26] Kanehisa M Goto S. Kegg: Kyoto encyclopedia of genes and genomes. Nucleic Acids Res. (2000) 28:27–30. doi: 10.1093/nar/28.1.27 10592173 PMC102409

[27] Szklarczyk D Gable AL Lyon D Junge A Wyder S Huerta-Cepas J. String V11: protein-protein association networks with increased coverage, supporting functional discovery in genome-wide experimental datasets. Nucleic Acids Res. (2019) 47:D607–D13. doi: 10.1093/nar/gky1131 PMC6323986 30476243

[28] Smoot ME Ono K Ruscheinski J Wang PL Ideker T. Cytoscape 2.8: new features for data integration and network visualization. Bioinformatics. (2011) 27:431–2. doi: 10.1093/bioinformatics/btq675 PMC3031041 21149340

[29] Chin CH Chen SH Wu HH Ho CW Ko MT Lin CY. Cytohubba: identifying hub objects and sub-networks from complex interactome. BMC Syst Biol. (2014) 8 Suppl 4:S11. doi: 10.1186/1752-0509-8-S4-S11 25521941 PMC4290687

[30] Li P Li T Zhang Z Dai X Zeng B Li Z. Corrigendum: bioinformatics and system biology approach to identify the influences among Covid-19, Ards and Sepsis. Front Immunol. (2023) 14:1236992. doi: 10.3389/fimmu.2023.1236992 37409126 PMC10319411

[31] Caramori G Casolari P Adcock I. Role of transcription factors in the pathogenesis of asthma and Copd. Cell Commun Adhes. (2013) 20:21–40. doi: 10.3109/15419061.2013.775257 23472830

[32] Rahman MR Islam T Shahjaman M Islam MR Lombardo SD Bramanti P. Discovering common pathogenetic processes between Covid-19 and diabetes mellitus by differential gene expression pattern analysis. Brief Bioinform. (2021) 22:bbab262. doi: 10.1093/bib/bbab262 34260684 PMC8344483

[33] Peng WF Bai F Shao K Shen LS Li HH Huang S. The key genes underlying pathophysiology association between the type 2-diabetic and colorectal cancer. J Cell Physiol. (2018) 233:8551–7. doi: 10.1002/jcp.26440 29319171

[34] Ahmed F Ansari JA Ansari ZE Alam Q Gan SH Kamal MA. A molecular bridge: connecting type 2 diabetes and Alzheimer's disease. CNS Neurol Disord Drug Targets. (2014) 13:312–21. doi: 10.2174/18715273113126660133 24059325

[35] Liu Q Xie W Wang Y Chen S Han J Wang L. Jak2/Stat1-mediated Hmgb1 translocation increases inflammation and cell death in a ventilator-induced lung injury model. Lab Invest. (2019) 99:1810–21. doi: 10.1038/s41374-019-0308-8 31467427

[36] Wang Y Li Y Zhang P Baker ST Wolfson MR Weiser JN. Regenerative therapy based on mirna-302 mimics for enhancing host recovery from pneumonia caused by streptococcus pneumoniae. Proc Natl Acad Sci USA. (2019) 116:8493–8. doi: 10.1073/pnas.1818522116 PMC6486708 30971494

[37] Lutz G Martin A Ursula B Johannes B Reinhard B Barbara G. Human immunodeficiency virus (Hiv). Transfus Med Hemother. (2016) 43:203–22. doi: 10.1159/000445852 PMC4924471 27403093

[38] Bertagnolio S Thwin SS Silva R Nagarajan S Jassat W Fowler R. Clinical Features of, and Risk Factors for, Severe or Fatal Covid-19 among People Living with Hiv Admitted to Hospital: Analysis of Data from the Who Global Clinical Platform of Covid-19. Lancet HIV. (2022) 9:e486–e95. doi: 10.1016/S2352-3018(22)00097-2 PMC9090268 35561704

[39] Ssentongo P Heilbrunn ES Ssentongo AE Advani S Chinchilli VM Nunez JJ. Epidemiology and outcomes of Covid-19 in Hiv-infected individuals: A systematic review and meta-analysis. Sci Rep. (2021) 11:6283. doi: 10.1038/s41598-021-85359-3 33737527 PMC7973415

[40] Varshney K Ghosh P Stiles H Iriowen R. Risk factors for Covid-19 mortality among people living with Hiv: A scoping review. AIDS Behav. (2022) 26:2256–65. doi: 10.1007/s10461-022-03578-9 PMC8756751 35024992

[41] Dong Z Yan Q Cao W Liu Z Wang X. Identification of key molecules in Covid-19 patients significantly correlated with clinical outcomes by analyzing transcriptomic data. Front Immunol. (2022) 13:930866. doi: 10.3389/fimmu.2022.930866 36072597 PMC9441550

[42] Wang Y Li J Zhang L Sun HX Zhang Z Xu J. Plasma cell-free Rna characteristics in Covid-19 patients. Genome Res. (2022) 32:228–41. doi: 10.1101/gr.276175.121 PMC8805721 35064006

[43] Zhou A Dong X Liu M Tang B. Comprehensive transcriptomic analysis identifies novel antiviral factors against influenza a virus infection. Front Immunol. (2021) 12:632798. doi: 10.3389/fimmu.2021.632798 34367124 PMC8337049

[44] Venkatachari NJ Jain S Walker L Bivalkar-Mehla S Chattopadhyay A Bar-Joseph Z. Transcriptome analyses identify key cellular factors associated with Hiv-1-associated neuropathogenesis in infected men. AIDS (London England). (2017) 31:623–33. doi: 10.1097/qad.0000000000001379 PMC5389669 28005686

[45] Kalil AC Thomas PG. Influenza virus-related critical illness: pathophysiology and epidemiology. Crit Care. (2019) 23:258. doi: 10.1186/s13054-019-2539-x 31324202 PMC6642581

[46] Bianco C Mohr I. Ribosome biogenesis restricts innate immune responses to virus infection and DNA. Elife. (2019) 8:e49551. doi: 10.7554/eLife.49551 31841110 PMC6934380

[47] Mohr I Sonenberg N. Host translation at the nexus of infection and immunity. Cell Host Microbe. (2012) 12:470–83. doi: 10.1016/j.chom.2012.09.006 PMC7104986 23084916

[48] Stern-Ginossar N Thompson SR Mathews MB Mohr I. Translational control in virus-infected cells. Cold Spring Harb Perspect Biol. (2019) 11:a033001. doi: 10.1101/cshperspect.a033001 29891561 PMC6396331

[49] Sun Z Ke L Zhao Q Qu J Hu Y Gao H. The use of bioinformatics methods to identify the effects of Sars-Cov-2 and influenza viruses on the regulation of gene expression in patients. Front Immunol. (2023) 14:1098688. doi: 10.3389/fimmu.2023.1098688 36911695 PMC9992716

[50] Li R Jiang X Zhang Y Wang S Chen X Yu X. Cyclin B2 overexpression in human hepatocellular carcinoma is associated with poor prognosis. Arch Med Res. (2019) 50:10–7. doi: 10.1016/j.arcmed.2019.03.003 31101236

[51] Zhang R Wei RL Du W Zhang LW Du T Geng YD. Long noncoding Rna enst00000413528 sponges microrna-593-5p to modulate human glioma growth via polo-like kinase 1. CNS Neurosci Ther. (2019) 25:842–54. doi: 10.1111/cns.13121 PMC6630009 30924320

[52] Dalvi PS Macheleidt IF Lim SY Meemboor S Müller M Eischeid-Scholz H. Lsd1 inhibition attenuates tumor growth by disrupting plk1 mitotic pathway. Mol Cancer Res MCR. (2019) 17:1326–37. doi: 10.1158/1541-7786.Mcr-18-0971 30760542

[53] Liu Y Shi Y Han R Liu C Qin X Li P. Signaling pathways of oxidative stress response: the potential therapeutic targets in gastric cancer. Front Immunol. (2023) 14:1139589. doi: 10.3389/fimmu.2023.1139589 37143652 PMC10151477

[54] Lisci M Barton PR Randzavola LO Ma CY Marchingo JM Cantrell DA. Mitochondrial translation is required for sustained killing by cytotoxic T cells. Sci (New York NY). (2021) 374:eabe9977. doi: 10.1126/science.abe9977 34648346

[55] Cao B Li XW Mao Y Wang J Lu HZ Chen YS. Clinical features of the initial cases of 2009 pandemic influenza a (H1n1) virus infection in China. N Engl J Med. (2009) 361:2507–17. doi: 10.1056/NEJMoa0906612 20007555

[56] Lim J Byun J Guk K Hwang SG Bae PK Jung J. Highly sensitive *in vitro* diagnostic system of pandemic influenza a (H1n1) virus infection with specific microrna as a biomarker. ACS Omega. (2019) 4:14560–8. doi: 10.1021/acsomega.9b01790 PMC6740188 31528810

[57] Xu J Zhou H Cheng Y Xiang G. Identifying potential signatures for atherosclerosis in the context of predictive, preventive, and personalized medicine using integrative bioinformatics approaches and machine-learning strategies. EPMA J. (2022) 13:433–49. doi: 10.1007/s13167-022-00289-y PMC9437201 36061826

[58] Gjorgjieva T Chaloemtoem A Shahin T Bayaraa O Dieng MM Alshaikh M. Systems genetics identifies Mirna-mediated regulation of host response in Covid-19. Hum Genomics. (2023) 17:49. doi: 10.1186/s40246-023-00494-4 37303042 PMC10257974

[59] Banaganapalli B Al-Rayes N Awan ZA Alsulaimany FA Alamri AS Elango R. Multilevel systems biology analysis of lung transcriptomics data identifies key Mirnas and potential Mirna target genes for Sars-Cov-2 infection. Comput Biol Med. (2021) 135:104570. doi: 10.1016/j.compbiomed.2021.104570 34157472 PMC8197616

[60] Vastrad B Vastrad C Tengli A. Bioinformatics analyses of significant genes, related pathways, and candidate diagnostic biomarkers and molecular targets in Sars-Cov-2/Covid-19. Gene Rep. (2020) 21:100956. doi: 10.1016/j.genrep.2020.100956 33553808 PMC7854084

[61] Mobley JA Molyvdas A Kojima K Ahmad I Jilling T Li JL. The Sars-Cov-2 spike S1 protein induces global proteomic changes in Atii-like rat L2 cells that are attenuated by hyaluronan. Am J Physiol Lung Cell Mol Physiol. (2023) 324:L413–L32. doi: 10.1152/ajplung.00282.2022 PMC10042596 36719087

[62] Haslbauer JD Savic Prince S Stalder AK Matter MS Zinner CP Jahn K. Differential gene expression of Sars-Cov-2 positive bronchoalveolar lavages: A case series. Pathobiology. (2023). doi: 10.1159/000532057 PMC10997241 37490884

[63] Woodson CM Kehn-Hall K. Examining the role of egr1 during viral infections. Front Microbiol. (2022) 13:1020220. doi: 10.3389/fmicb.2022.1020220 36338037 PMC9634628

[64] Cao L Liu Y Ma B Yi B Sun J. Discovery of natural multi-targets neuraminidase inhibitor glycosides compounds against influenza a virus through network pharmacology, virtual screening, molecular dynamics simulation, and *in vitro* experiment. Chem Biol Drug Des. (2023) 103:e14359. doi: 10.1111/cbdd.14359 37743355

[65] Zhang F Lin X Yang X Lu G Zhang Q Zhang C. Microrna-132-3p suppresses type I Ifn response through targeting Irf1 to facilitate H1n1 influenza a virus infection. Biosci Rep. (2019) 39:BSR20192769. doi: 10.1042/BSR20192769 31746331 PMC6904772

[66] Basu D Chavda VP Mehta AA. Therapeutics for Covid-19 and post Covid-19 complications: an update. Curr Res Pharmacol Drug Discov. (2022) 3:100086. doi: 10.1016/j.crphar.2022.100086 35136858 PMC8813675

[67] Cabrera-Munoz E Hernandez-Hernandez OT Camacho-Arroyo I. Role of estradiol and progesterone in Hiv susceptibility and disease progression. Mini Rev Med Chem. (2012) 12:1049–54. doi: 10.2174/138955712802762185 22827217

[68] Seeland U Coluzzi F Simmaco M Mura C Bourne PE Heiland M. Evidence for treatment with estradiol for women with Sars-Cov-2 infection. BMC Med. (2020) 18:369. doi: 10.1186/s12916-020-01851-z 33234138 PMC7685778

[69] Heidari S Mohammadi S Fathi M Cigary S Alisamir M Mirkarimi M. Association of vitamin D status with Covid-19 disease severity in pediatric patients: A retrospective observational study. Health Sci Rep. (2022) 5:e569. doi: 10.1002/hsr2.569 35415272 PMC8987118

[70] Callaghan PJ Rybakovsky E Ferrick B Thomas S Mullin JM. Retinoic acid improves baseline barrier function and attenuates Tnf-Alpha-induced barrier leak in human bronchial epithelial cell culture model, 16hbe 14o. PloS One. (2020) 15:e0242536. doi: 10.1371/journal.pone.0242536 33301441 PMC7728186

[71] Sarohan AR. Covid-19: endogenous retinoic acid theory and retinoic acid depletion syndrome. Med Hypotheses. (2020) 144:110250. doi: 10.1016/j.mehy.2020.110250 33254555 PMC7481114

[72] Yuan L Zhu H Wu K Zhou M Ma J Chen R. Female sex hormone, progesterone, ameliorates the severity of Sars-Cov-2-caused pneumonia in the Syrian hamster model. Signal Transduct Target Ther. (2022) 7:47. doi: 10.1038/s41392-021-00860-5 35165265 PMC8844354

[73] Ahmad SI. 5-fluorouracil in combination with deoxyribonucleosides and deoxyribose as possible therapeutic options for the coronavirus, Covid-19 infection. Med Hypotheses. (2020) 142:109754. doi: 10.1016/j.mehy.2020.109754 32438240 PMC7194918

[74] Ganjei Z Faraji Dana H Ebrahimi-Dehkordi S Alidoust F Bahmani K. Methotrexate as a safe immunosuppressive agent during the Covid-19 pandemic. Int Immunopharmacol. (2021) 101:108324. doi: 10.1016/j.intimp.2021.108324 34731780 PMC8556580

[75] Skevaki C Karsonova A Karaulov A Xie M Renz H. Asthma-associated risk for Covid-19 development. J Allergy Clin Immunol. (2020) 146:1295–301. doi: 10.1016/j.jaci.2020.09.017 PMC7834224 33002516

[76] Oreskovic NM Kinane TB Aryee E Kuhlthau KA Perrin JM. The unexpected risks of Covid-19 on asthma control in children. J Allergy Clin Immunol Pract. (2020) 8:2489–91. doi: 10.1016/j.jaip.2020.05.027 PMC7263244 32497662

[77] Fang L Karakiulakis G Roth M. Are patients with hypertension and diabetes mellitus at increased risk for Covid-19 infection? Lancet Respir Med. (2020) 8:e21. doi: 10.1016/s2213-2600(20)30116-8 32171062 PMC7118626

[78] Hu H Tang N Zhang F Li L Li L. Bioinformatics and system biology approach to identify the influences of Covid-19 on rheumatoid arthritis. Front Immunol. (2022) 13:860676. doi: 10.3389/fimmu.2022.860676 35464423 PMC9021444

[79] Pathania AS Prathipati P Abdul BA Chava S Katta SS Gupta SC. Covid-19 and cancer comorbidity: therapeutic opportunities and challenges. Theranostics. (2021) 11:731–53. doi: 10.7150/thno.51471 PMC7738845 33391502

[80] Sansoè G Aragno M Wong F. Covid-19 and liver cirrhosis: focus on the nonclassical renin-angiotensin system and implications for therapy. Hepatol (Baltimore Md). (2021) 74:1074–80. doi: 10.1002/hep.31728 PMC8013494 33524188

[81] Mossmann D Muller C Park S Ryback B Colombi M Ritter N. Arginine reprograms metabolism in liver cancer via Rbm39. Cell. (2023) 186:5068–83.e23. doi: 10.1016/j.cell.2023.09.011 37804830 PMC10642370

[82] Wu SJ Tang JL Lin CT Kuo YY Li LY Tseng MH. Clinical implications of U2af1 mutation in patients with myelodysplastic syndrome and its stability during disease progression. Am J Hematol. (2013) 88:E277–82. doi: 10.1002/ajh.23541 23861105

[83] Aloufi N Haidar Z Ding J Nair P Benedetti A Eidelman DH. Role of human antigen R (Hur) in the regulation of pulmonary Ace2 expression. Cells. (2021) 11:22. doi: 10.3390/cells11010022 35011584 PMC8750694

[84] Frasco MA Karim R Van Den Berg D Watanabe RM Anastos K Cohen M. Antiretroviral therapy modifies the genetic effect of known type 2 diabetes-associated risk variants in Hiv-infected women. AIDS (London England). (2014) 28:1815–23. doi: 10.1097/QAD.0000000000000366 PMC4269472 24932614

[85] Zhou Z Lu Y Gu Z Sun Q Fang W Yan W. Hnrnpa2b1 as a potential therapeutic target for thymic epithelial tumor recurrence: an integrative network analysis. Comput Biol Med. (2023) 155:106665. doi: 10.1016/j.compbiomed.2023.106665 36791552

