# Supplementary material for: Bioinformatics and system biology approach to identify the influences among COVID-19, influenza, and HIV on the regulation of gene expression
Source: Front Immunol. 2024 Mar 27;15:1369311. doi: 10.3389/fimmu.2024.1369311 (PMC11004287; doi:10.3389/fimmu.2024.1369311)
Supplement: Supplementary file 1 [file Table_1.docx]

Supplementary Material

Supplementary Table 1 Abbreviation and full name comparison table

| COVID-19 | severe coronavirus disease 2019 |
| --- | --- |
| SARS-CoV-2 | severe acute respiratory syndrome coronavirus 2 |
| AIDS | Acquired Immunodeficiency Syndrome |
| HIV | human immunodeficiency virus |
| NCBI | National Center for Biotechnology Information |
| GEO | Gene Expression Omnibus |
| DEGs | differentially expressed genes |
| ROC | receiver operating characteristic |
| AUC | area under curve |
| ssGSEA | single-sample Gene Set Enrichment Analysis |
| GO | Gene Ontology |
| BP | biological process |
| CC | cell composition |
| MF | molecular function |
| KEGG | Kyoto Encyclopedia of Genes and Genomes |
| GSEA | Gene Set Enrichment Analysis |
| FDR | False Discovery Rate |
| PPIs | Protein-Protein Interaction Networks |
| miRNAs | microRNAs |
| TFs | transcription factors |
| RBPs | RNA-binding proteins |
| qPCR | quantitative polymerase chain reaction |

Supplementary Table 2 Primer sequences for qPCR

| Gene | Direction | Primer sequence (5' →  3') |
| --- | --- | --- |
| IFI44 | Forward | GGTGGGCACTAATACAACTGG |
|  | Reverse | CACACAGAATAAACGGCAGGTA |
| IFIT3 | Forward | AGAAAAGGTGACCTAGACAAAGC |
|  | Reverse | CCTTGTAGCAGCACCCAATCT |
| OAS1 | Forward | TGGAAGCCTGTCAAAGAGAGAGA |
|  | Reverse | GGGTTAGGTTTATAGCCGCCAGT |
| EIF2AK2 | Forward | TGGAAAGCGAACAAGGAGTAAG |
|  | Reverse | CCAAAGCGTAGAGGTCCACTT |
| IFI27 | Forward | TGCTCTCACCTCATCAGCAGT |
|  | Reverse | CACAACTCCTCCAATCACAACT |
| OASL | Forward | CCATTGTGCCTGCCTACAGAG |
|  | Reverse | CTTCAGCTTAGTTGGCCGATG |
| EPSTI1 | Forward | ATGAAGGCAATTCAGAGAGAGAA |
|  | Reverse | GCTATCAAGGTGTATGCACTTGT |
| IFI44L | Forward | ACAGAGCCAAATGATTCCCTATG |
|  | Reverse | TCGATAAACGACACACCAGTTG |
| RSAD2 | Forward | CAAAAGCTGAGGAGGTGGTGTAG |
|  | Reverse | GGACAGGGTTTAGTGCTTTGATC |
| GAPDH | Forward | GAAGCTTGTCATCAATGGAAATC |
|  | Reverse | CAGAGATGATGACCCTTTTGG |
